# Measuring fidelity to manualised peer support for people with severe mental health conditions: development and psychometric evaluation of the UPSIDES fidelity scale

**DOI:** 10.1186/s12888-024-06081-8

**Published:** 2024-10-11

**Authors:** Ramona Hiltensperger, Yasuhiro Kotera, Philip Wolf, Rebecca Nixdorf, Ashleigh Charles, Marianne Farkas, Alina Grayzman, Jasmine Kalha, Palak Korde, Candelaria Mahlke, Galia Moran, Richard Mpango, Rachel Mtei, Grace Ryan, Donat Shamba, Lisa Wenzel, Mike Slade, Bernd Puschner

**Affiliations:** 1 Department of Psychiatry II, Ulm University, Ulm, Germany; 2 School of Health Sciences, Institute of Mental Health, University of Nottingham, Nottingham, UK; 3 Department of Psychiatry, University Medical Center Hamburg-Eppendorf, Hamburg, Germany; 4 Center for Psychiatric Rehabilitation, Boston University, Boston, USA; 5 Department of Social Work, Ben Gurion University of the Negev, Be’er Sheva, Israel; 6 Centre for Mental Health Law & Policy, Indian Law Society, Pune, India; 7 Butabika National Referral Hospital, Kampala, Uganda; 8 Department of Mental Health, School of Health Sciences, Soroti University, Soroti, Uganda; 9 MRC/UVRI and LSHTM Uganda Research Unit, Entebbe, Uganda; 10 Ifakara Health Institute, Dar es Salaam, Tanzania; 11 London School of Hygiene and Tropical Medicine, Centre of Global Mental Health, London, UK; 12 Faculty of Nursing and Health Sciences, Health and Community Participation Division, Nord University, Postbox 474, Namsos, 7801 Norway

**Keywords:** Peer support, Fidelity, Scale development, Psychometric evaluation, Recovery, Lived experience

## Abstract

**Background:**

Peer support workers provide support for people experiencing mental health conditions based on their own lived experience of mental health problems. Assessing fidelity to core ingredients of peer support is vital for successful implementation and intervention delivery. Modifications to its implementation are needed when scaling up to different socio-economic settings, raising further uncertainty about fidelity. As part of a large multi-centre study on peer support called Using Peer Support In Developing Empowering Mental Health Services (UPSIDES), we developed and evaluated the psychometric properties of the UPSIDES Fidelity Scale.

**Methods:**

We constructed the fidelity scale based on an initial item pool developed through international expert consultation and iterative feedback. Scale refinement involved site-level expert consultation and translation, resulting in a service user-rated 28-item version and a peer support worker-rated 21-item version assessing receipt, engagement, enactment, competence, communication and peer support-specific components. Both versions are available in six languages: English, German, Luganda, Kiswahili, Hebrew and Gujarati. The scale was then evaluated at six study sites across five countries, with peer support workers and their clients completing their respective ratings four and eight months after initial peer support worker contact. Psychometric evaluation included analysis of internal consistency, construct validity and criterion validity.

**Results:**

For the 315 participants, item statistics showed a skewed distribution of fidelity values but no restriction of range. Internal consistency was adequate (range α = 0.675 to 0.969) for total scores and all subscales in both versions. Confirmatory factor analysis indicated acceptable fit of the proposed factor structure for the service user version (χ2/df = 2.746; RMSEA = 0.084) and moderate fit for the peer support worker version (χ2/df = 3.087; RMSEA = 0.093). Both versions showed significant correlations with external criteria: number of peer support sessions; perceived recovery orientation of the intervention; and severity of illness.

**Conclusions:**

The scale demonstrates good reliability, construct and criterion validity, making it a pragmatic and psychometrically acceptable measure for assessing fidelity to a manualised peer support worker intervention. Recommendations for use, along with research and practical implications, are addressed. As validated, multi-lingual tool that adapts to diverse settings this scale is uniquely positioned for global application.

**Trial registration:**

ISRCTN, ISRCTN26008944. Registered on 30 October 2019.

**Supplementary Information:**

The online version contains supplementary material available at 10.1186/s12888-024-06081-8.

## Background

Peer support is an established intervention to promote mental health recovery and empowerment in many countries around the world [1, 2]. Trained peer support workers (PSWs) can provide support and hope, and foster empowerment of people facing mental health difficulties, based on their own lived experience of mental health conditions and recovery [3, 4]. Ensuring fidelity is important to (a) maintain the quality of complex mental health interventions and (b) developing a robust evidence base for any practice [5], as is also the case for peer support interventions [6]. In order to assess and ensure the fidelity of peer support interventions there is a need for psychometrically sound scales which measure fidelity to manualised peer support [7]. This becomes especially relevant when a peer support intervention is scaled up to various social, cultural and economic settings, as in multicentre global health projects. The quality of service delivery of a manualised intervention often fluctuates across different practitioners, sites of delivery, levels of expertise and cultural contexts [8]. Research suggests that fluctuations in manual fidelity across service providers and settings impact successful implementation of psychosocial interventions [9–11]. Fidelity assessment helps to mitigate the risk of these inconsistencies [12] and supports successful implementation of complex interventions. There is a wide range of approaches to fidelity measurement in a variety of contexts in mental health services research [13]. Fidelity measures typically focus on the interventions’ critical elements and are mostly limited to a single perspective, usually either an external third observer or the service provider [14–16]. For peer support for adults with mental health conditions, we identified two fidelity measures, both avaible in English only. One is a PSW-rated questionnaire (Chinman et al., 2016), piloted with 12 peer specialists and supervisors. Results from cognitive interviews indicate usability of this scale, but no psychometric evaluation has been published. A more recent measure [17] is conceptualised as a fidelity index, assessing fidelity of the overall service through semi-structured interviews with PSWs, their supervisors and people receiving peer support. It was developed based on expert panels and a peer support framework and has good psychometric properties. Despite its strengths, this measure is very detailed and requires substantial resources for training of interviewers and interviewing multiple participants which is burdensome and limits its use for low-resource settings. Further, it was conceptualised for measuring fidelity of peer support in a high-income country context. There is not yet any information available on its applicability to contexts outside the UK.

Overall, there is a dearth of evidence on the assessment of fidelity to manualised peer support interventions, especially with regard to measuring fidelity (a) on an individual rather than overall service level, (b) including the view of recipients of service, (c) in a pragmatic and resource-efficient way and (d) for multicentre trials with intervention sites in different socio-economic settings. Further, there is a need for multi-language versions to support global implementation of peer support. Despite existing research, there remains a significant gap in validated, culturally adaptable fidelity scales for peer support, a gap this study aims to address by developing a scale that is both multi-lingual and applicable across diverse settings.

The aim of this study was to develop and evaluate a brief and easy-to-use measure to assess fidelity of UPSIDES peer support in six languages. The objectives were: (1) to construct an initial version of the scale based on existing literature and UPSIDES principles of peer support, (2) to refine the scale with input from international key stakeholders and experts from all study sites and (3) to evaluate the psychometric properties of the scale.

## Methods

This study was conducted as part of the Using Peer Support In Developing Empowering Mental Health Services (UPSIDES) project (www.upsides.org). UPSIDES aims to replicate, scale-up and evaluate a peer support intervention for people with severe mental health conditions in high-, middle- and low-resource settings [18, 19]. The UPSIDES intervention is desgined to be delivered by peer support workers with lived experience of mental health conditions and focuses on social inclusion and recovery support for people with serious mental health conditions. The intervention can be implemented in various inpatient and outpatient settings and provides i.a. practical support and conflict mediation, and promotes recovery-orientation within mental health teams [20]. As part of the UPSIDES-RCT [19], data werecollected between 2020 and 2022 at six UPSIDES collaborating institutions in Germany, Uganda, Tanzania, Israel and India^1^. The UPSIDES RCT used a wait-list design, with four measurement points (T0 = baseline; T1 = 4 months; T2 = 8 months; T3 = 12 months). Participants allocated to the intervention group additionally received a minimum of three sessions of UPSIDES peer support for up to six months after baseline assessment. PSWs met with their clients either one-to-one (Ulm, Hamburg, Butabika, Dar es Salaam, Pune), or in a small group format (Be’er Sheva). Two sites (Hamburg and Dar es Salaam) offered optional group meetings in addition to their one-to-one meetings. Weekly or biweekly meetings were recommended, but frequency varied intentionally depending on the needs of the service users (SUs), PSWs and study sites. Through the UPSIDES training, the PSWs learned how to use their experiential knowledge to support their clients and to work according to the nine UPSIDES peer support principles: mutual, reciprocal, non-directive, recovery-focused, empowerment, strengths-based, inclusive & community focused, trialogue, safe [21, 22]. All study sites have implemented the intervention based on common implementation guidance, for example regarding recruitment of PSWs, quality assurance and stakeholder engagement [12, 23].

The UPSIDES Fidelity Scale was developed in three stages, based on recommendations by Bond & Drake [24]. In Stage 1 (Construction), we defined the content of the scale, more specifically its purpose and key principles. We synthesised input from existing literature and field versions of the UPSIDES training manual [21] and the UPSIDES implementation manual [23] to create the initial version of the scale. Fidelity to the UPSIDES intervention was defined as delivering the intervention in line with the UPSIDES Training Manual and Workbook, i.e. the extent to which central components of UPSIDES peer support have been delivered in the peer support sessions. Two versions of a self-report measure with a five-point Likert-scale were developed, one for PSWs and another for SUs to assess fidelity from two different perspectives. The measurement of fidelity from the perspectives of both providers and recipients of peer support is an important and innovative contribution to fidelity research in the field. This approach has been developed for other recovery-oriented interventions [25], but here for the first time for peer support. Part 1 of the scale evaluates implementation aspects (receipt, engagement, enactment, competence and communication), while Part 2 focuses on peer support specifics, derived from the nine principles of peer support of the UPSIDES conceptual framework. In Stage 2 (Refinement), we performed an expert consultation to obtain input from international key stakeholders from all study sites to refine the scale. Nine key informants rated item importance, leading to the removal of some items to reduce length resulting in a 21-item version for PSWs and a 28-item version for SUs. The item selection was guided by theoretical constructs from existing peer support literature, emphasizing elements crucial for maintaining intervention fidelity in varied cultural and linguistic contexts. The wording of several items was improved for clarity and precision. Final language editing and translation into five languages were conducted following the UPSIDES proportionate translation methodology [26]. In Stage 3 (Psychometric evaluation) we explored the following psychometric properties of the scale: reliability via internal consistency, construct validity via confirmatory factor analysis and criterion validity in relation to external criteria. The sample information and detailed description of the procedures for Stages 1 and 2, for example rationales regarding the definition of the scale content, can be found in Additional file 1. Methods for stage 3 are described in the following. The final scale is provided in English, German, Luganda, Swahili, Hebrew and Gujarati in Additional file 2.

### Participants

For stage 3 of the scale development, the psychometric evaluation of the scale, data of PSWs and SUs who participated in the UPSIDES RCT were analysed. The inclusion criteria for SU participants were: adult age (18–60 years) at intake; mental disorder of any kind as main diagnosis established by case notes, staff communication or self-label; presence of severe mental illness (Threshold Assessment Grid, TAG [19] ≥ 5 points and illness duration ≥ 2 years); sufficient command of the host country’s language; capable of giving informed consent. This study only analysed data of intervention group participants who received at least three sessions of peer support over a maximum period of six months in either a one-to-one or group-setting. Participants who received less than three sessions were excluded from the dataset.

The inclusion criteria for PSWs were: adults (age 18–60 years) who have experienced a mental illness and who have been stable or out of hospital for at least three months. PSWs have progressed in their recovery and are using their personal experiences, along with UPSIDES training and supervision, to facilitate, guide and mentor another person’s recovery journey. Throughout the intervention all PSWs received supervision at regular intervals by mental health professionals, experienced PSWs and via mutual support groups [20, 21]. More details on the UPSIDES RCT and the intervention are described elsewhere [19–21].

### Procedures

Sociodemographic information was collected at baseline. No data regarding type of mental illness were collected for PSWs at baseline as this would have interfered with local recruitment guidelines. Each PSW and associated SU completed UFS-P or UFS-S respectively, at regular fidelity audit points at T1 (4 months after the start of the intervention) and T2 (8 months after the start of the intervention). Mean fidelity scores were calculated if at least 80% of all questions were answered. Participant data were included in the analyses if at least one fidelity questionnaire (UFS-S or UFS-P) was completed with more than 80% of all items answered. The number of peer support sessions was assessed by PSWs and research workers via UPSIDES Monitoring and Evaluation forms completed over the course of the intervention. The timing of the sessions afforded a lot of flexibility accounting for the broad variety at different international sites. After completion of the study, it became apparent that in many cases the interventions were already finished before the audit at T1; across all sites, the mean duration of the intervention was M = 126.46 (SD = 77.27) days, on average participants received 2.2 sessions per month (SD = 1.5). Subsequently, for most SUs the audit at the T2 assessment was several months after their last intervention session, which may lead to recall bias. Therefore, we have decided for all further psychometric analyses to only analyse T1 data, exclusively. At the Israeli study site, due to the local implementation strategy (peer support groups facilitated by PSW dyads) there were two PSWs per SU; subsequently, two UFS-P questionnaires per SU and timepoint were available. Only one was included in the data analyses. The decision on inclusion for data analysis was made individually at random during data entry before any analyses were conducted.

### Measures

Both, SUs and PSWs filled in the UPSIDES baseline questionnaire to provide sociodemographic information and provided fidelity data via the respective fidelity scale version at T1 and T2. To evaluate criterion validity of the scales, fidelity scores were examined in relation to three external criteria: SUs’ recovery experiences, SUs’ severity of mental health conditions and the number of intervention sessions. The former two were measured with established questionnaires. The SUs’ experiences of the support they receive from their PSW with regard to their recovery were assessed with the Brief INSPIRE, a five-item short version of the INSPIRE questionnaire [27]. The measure is a SU-rated experience measure based on five key recovery processes — Connectedness, Hope, Identity, Meaning and Empowerment (the CHIME Framework). SUs were asked to answer on a five-point Likert scale (ranging from “Not at all” to “Very much”) how well the respective PSW helps with their recovery on the five key recovery processes. The severity of illness of SUs was assessed with the Threshold Assessment Grid (TAG) [28] at baseline. The TAG is a seven-item staff-rated assessment of the severity of a SU’s mental health conditions comprising seven domains (intentional self-harm, unintentional self-harm, risk from others, risk to others, survival, psychological, social). Each item is answered on a five-point Likert scale (ranging from “disagree” to “agree”). The test-retest reliability of TAG is high (alpha = 0.87) [29]. In UPSIDES, the TAG score was rated by research staff interviewing study participants.

### Analysis

Internal consistency was assessed using Cronbach’s Alpha. Construct validity was assessed using confirmatory factor analysis (CFA). We hypothesised that both the SU and the PSW version of the scale consist of two factors: common aspects of fidelity (Part 1, implementation) and aspects specific to UPSIDES peer support (Part 2, active ingredients). Based on the theoretical foundation of the scale, a factor model of higher order was specified a priori and tested in which the first factor (“implementation”) was divided into subscales (four domains in the SU version, three domains in the PSW version). It was tested against a more parsimonious two-factor model that does not contain the subscales and only consists of the two factors “implementation” and “active ingredients”. We conducted the CFA using case-wise maximum likelihood estimation. Subsequently, standard errors were computed with the observed information matrix, not the expected matrix, and all available data were used. Several model fit indices were used to test the adequacy of the proposed factorial structure and comparative fit of the two models. The model fit was evaluated according to established guidelines by Schermelleh-Engel et al. and Hu & Bentler [30, 31]. The scale’s criterion validity was examined by analysing correlations between mean scores on the fidelity scale and three external criteria, see above [32]. All statistical analyses were conducted with SPSS Version 28, except for the CFA which was computed using R Version 4.3.1 and the R package “lavaan”. Graphs were drawn using the R package “semPlot”.

## Results

Fidelity data for Stage 3 (Psychometric evaluation) were obtained from 257 SUs and 58 PSWs. The sociodemographic data of the participants are presented in Table 1.

**Table 1:** Sociodemographic characteristics at baseline

Baseline characteristics	Service users	Peer support workers
	*N* = 257	*N* = 58
Age M (SD)	37.93 (11.08)	39.54
Gender n (%)		
Female	147 (57.2)	35 (60.3)
Male	109 (42.4)	23 (39.7)
Diverse	1 (0.4)	0
Marital status n (%)		
Single/Unmarried	147 (57.2)	39 (67.2)
Married	71 (27.6)	13 (22.4)
Seperated/Divorced	36 (14.0)	6 (10.3)
Widowed	2 (0.8)	5 (10.9)
Other	1 (0.4)	0
Type of mental illness n (%)		
Psychotic disorder	81 (31.5)	n/a
Bipolar disorder	73 (28.4)	n/a
Depressive disorder	73 (28.4)	n/a
Anxiety & PTSD	12 (4.7)	n/a
Personality disorder	9 (3.5)	n/a
Other	8 (3.1)	n/a
Highest educational level n (%)		
Primary or less	37 (14.4)	0
Secondary	153 (59.5)	22 (37.9)
Tertiary / further	56 (21.8)	32 (55.2)
Other general education	0	1 (5.2)
No formal education	8 (3.1)	0
Not known	2 (0.8)	3 (1.7)
Employment n (%)		
Paid or self-employed	52 (20.2)	32 (55.2)
Voluntary employment	11 (4.3)	7 (12.1)
Sheltered employment	25 (9.7)	1 (1.7)
Unemployed	109 (42.4)	4 (6.9)
Student	16 (6.2)	3 (5.2)
Housewife/Husband	14 (5.4)	2 (3.4)
Retired	12 (4.7)	4 (6.9)
Other	18 (7.0)	5 (8.6)
Study site n (%)		
Be’er Sheva, Israel	39 (15.2)	17 (29.3)
Kampala, Uganda	63 (24.5)	10 (17.2)
Dar es Salaam, Tanzania	41 (16.0)	10 (17.2)
Ahmedabad, India	43 (16.7)	4 (6.9)
Hamburg, Germany	43 (16.7)	10 (17.2)
Ulm, Germany	28 (10.9)	7 (12.1)

The descriptive statistics of mean fidelity scores at timepoints t1 and t2 are shown in Table 2.

**Table 2:** Descriptive statistics of mean fidelity scores at timepoints t1 and t2

	UFS-S Mean t1	UFS-S Mean t2	UFS-*P* Mean t1	UFS-*P* Mean t2
N	244	232	239	221
M (SD)	4.09 (0.75)	4.16 (0.83)	4.09 (0.60)	4.06 (0.70)
Minimum	1.18	1.00	2.00	1.44
Maximum	5.00	5.00	5.00	5.00
Skewness (SE)	− 0.80 (0.16)	-1.06 (0.16)	− 0.72 (0.16)	-1.15 (0.16)
Kurtosis (SE)	0.12 (0.31)	0.90 (0.32)	0.38 (0.31)	1.33 (0.33)

*Note* UFS-S = UPSIDES fidelity scale service user version, UFS-P = UPSIDES fidelity scale peer support worker version, M = mean, SD = standard deviation, SE = standard error

Mean scores of UFS-S and UFS-P across both timepoints ranged from 4.06 to 4.16. Participants used the entire scale with minimum and maximum means ranging from 1 to 5, with no evidence for floor or ceiling effects. The distribution of values was skewed to the right as indicated by negative skewness throughout both SU and PSW version and both time points and, at T2, had high positive kurtosis values.

### Reliability - internal consistency analysis

Cronbach’s alpha of the total scale was α = 0.969 for UFS-S and α = 0.922 for UFS-P. Cronbach’s alpha for subscales ranged from α = 0.675 to α = 0.954. All correlations of the individual items with their respective subscale are shown in Additional file 3.

### Construct validity - confirmatory factor analysis

The results of the confirmatory factor analyses can be found in Table 3. The model with factor 1 (implementation) being a factor of higher order showed better goodness of fit indices as compared to the two-factor model for both UFS-S and UFS-P. Significantly lower values of both AIC and BIC further confirmed the superiority of the first model despite the losses in parsimony (χ^2^ difference test *p* < .001). Overall, the CFA for the higher order model suggests acceptable fit of the proposed factor structure for UFS-S and moderate fit for UFS-P.

**Table 3:** Results of the confirmatory factor analyses for UFS-S and UFS-P and common goodness of fit threshold criteria

		M1: Higher-order model	M2: Two-factor model	Model fit thresholds (Hu & Bentler, 1999; Schermelleh-Engel et al., 2003)
UFS-S *N* = 248	χ^2^/df	2.746	3.064	2–3 good, 3–5 permissible
	CFI	0.893	0.872	> 0.95 good, > 0.90 permissible
	TLI	0.883	0.861	> 0.95 good, > 0.90 permissible
	RMSEA	0.084	0.091	< 0.05 good, < 0.05-0.10 permissible
	SRMR	0.049	0.050	< 0.05 good, < 0.05-0.10 permissible
	AIC	14711.378	14825.334***	comparative index
	BIC	15024.073	15123.976***	comparative index
UFS-P *N* = 243	χ^2^/df	3.087	3.746	2–3 good, 3–5 permissible
	CFI	0.849	0.798	> 0.95 good, > 0.90 permissible
	TLI	0.828	0.774	> 0.95 good, > 0.90 permissible
	RMSEA	0.093	0.106	< 0.05 good, < 0.05-0.10 permissible
	SRMR	0.063	0.072	< 0.05 good, < 0.05-0.10 permissible
	AIC	12159.421	12286.566***	comparative index
	BIC	12393.457	12510.122***	comparative index

*Notes* UFS-S = UPSIDES fidelity scale service user version, UFS-P = UPSIDES fidelity scale peer support worker version, M1 = model with two factors, factor implementation as factor of higher order, divided into sub-categories. Sub-categories in UFS-S: receipt, engagement, enactment, competence. Sub-categories in UFS-P: receipt, competence, communication. M2 = model with two factors (implementation and active ingredients), no sub-categories. χ2 = Chi-square index, df = Degrees of freedom, CFI = Comparative fit index, TLI = Tucker-Lewis index, RMSEA = Root mean square error of approximation, SRMR = Standardised root mean square residual. AIC = Aikaike information criterion, BIC = Bayesian information criterion. *** Comparison of AIC and BIC Model 1 vs. Model 2: χ2 difference test *p* < .001

Closer inspection of the higher-order factor structure revealed similar factor loadings of items of one subscale and high positive correlations between main factors (*r* = .97 for UFS-S; *r* = .95 for UFS-P). All factor loadings, correlations between factors and residuals for UFS-S and UFS-P are shown in Additional file 4.

### Criterion validity — relations with external criteria

The fidelity scale’s correlations with the three external criteria are presented in Table 4. Mean fidelity scores were moderately to highly positively correlated with number of sessions and Brief-INSPIRE score, and negatively correlated with the TAG score. Overall, correlations with external criteria were higher for UFS-S than UFS-P.

**Table 4:** Correlations of mean fidelity scores with external criteria

External criteria	Mean UFS-S	Mean UFS-*P*
Number of intervention sessions	0.411**	0.287**
Mean INSPIRE	0.761**	0.318**
TAG Score	− 0.153*	− 0.011

*Note* UFS-S = UPSIDES fidelity scale service user version, UFS-P = UPSIDES fidelity scale peer support worker version, N_SU_ = 244, N_PSW_ = 234. ** significant at 0.05 level, * significant at 0.01 level

## Discussion

This paper describes the development and psychometric evaluation of a SU- and PSW-rated fidelity scale for manualised peer support following a three-step model [24]. Its psychometric properties ( moderate variability of fidelity scores, slight skew towards the upper end of the scales, and high levels of internal consistency) are in line with findings for other fidelity measures [14, 17, 24, 33]. In two cases (item 8 of the UFS-S and item 9 of the UFS-P), the deletion of the item would have led to a small increase in the Cronbach’s alpha values. Since internal consistency was already high and removing the items would have resulted in only a small increase in Cronbach’s alpha values, we decided to retain the items in order to further reflect the underlying theoretical concepts. Based on this however we derive that the development and piloting of a short version of the scale is an implication for future research. The confirmatory factor analyses suggest an acceptable fit of the proposed factor structure for the SU data and a moderate fit for the PSW data which is comparable to fit indices of other similar instruments [16, 33, 34]. Regarding criterion validity, moderate to strong positive correlations between fidelity scores, peer support sessions, and recovery experiences and weak negative correlations with illness severity were found [35], aligning with our a-priori hypotheses.

We anticipated a potential decrease in fidelity associated with health condition severity due to various factors. For example, it is possible that the mode of delivery did not allow for additional more intensive peer support for individuals with increased severity of mental health conditions. In addition, certain elements of peer support interventions may be less important during periods of high severity, which could lead to lower fidelity to these components. Considerations such as reciprocity may also play a role, with peer supporters often utilizing their expertise gained through experience. As in any other field, expertise and practice are interrelated, and this dynamic could affect fidelity ratings. In particular, in newly established peer support programmes compared to more established services, there are greater resource needs and less experienced staff members whose ability to provide quality support to people with particularly severe mental health conditions has yet to be consolidated.

### Strengths and limitations

A limitation of this study is that the development of the scale was not based on a systematic literature review, which may have led to some existing scales not being found in our search and thus not being included in the development of the initial item pool. Further, assessing fidelity via self-report may have affected the validity and accuracy of the data due to its high potential for social- and self-desirability biases [36, 37]. However, since UPSIDES relies heavily on the rapport between peers and PSWs, having an observer (e.g. supervisor) present during interactions or recording sessions for later assessment would compromise the intervention’s quality and is not acceptable due to the personal and private nature of the information shared.

Another caveat might be that service user ratings of fidelity might differ from those of peer support workers because only the latter have received the training and know the manual. To cater to these differences, the service user version of the UPSIDES fidelity scale has been constructed in a way so that persons without this previous knowledge can give meaningful information, and a central part of this paper is the comparison of ratings from both perspectives. We believe that, in addition to peer support providers, service users are an important source to measure fidelity. Regarding the psychometric evaluation of the scale, the high Cronbach’s alpha values and the high correlation between the subscales implementation and active ingredients are indicators of redundancy [38]. However, when Cronbach’s alphas were calculated individually for the subscales, the values were acceptable and did not show signs of redundancy within the subscales. Taken together, the high internal consistency of the total scale and the strong correlation between the factors support the use of the mean fidelity score across all subscales for assessing peer support fidelity [34]. Another limitation is the differences in sample size for the PSW-rated fidelity scores, especially the comparably low sample size of four PSWs in Ahmedabad, India. Therefore, only limited conclusions can be drawn for the Gujarati PSW-rated version of the scale. While the scale demonstrated adequate reliability, the range of alpha values observed suggests a need for further investigation into specific items that may perform differently across cultural settings, potentially affecting the scale’s overall reliability. Additionally, the methodological approaches, including the sampling method and response rate, may limit the generalisability of the findings. Different cultural responses to self-reporting may also influence the data, necessitating cautious interpretation across contexts. The translation process could have been further strengthened by incorporating a formal back-translation process.

There are several strengths of our study. Firstly, we could confirm that measuring fidelity via self-report and collecting information directly from SUs and PSWs was most pragmatic and feasible for our purposes, and it also provided insights from the perspective of both providers and intervention recipients. The multi-perspective nature of the measure allows future research to compare SUs’ and PSWs’ perspectives. Further, our fidelity measure is theoretically-based (i.e. on PSW components in the UPSIDES manual and other existing literature [13, 15, 39]) and is available in six language versions (English, German, Luganda, Swahili, Hebrew and Gujarati) which all have been successfully used in a global health trial.

### Research implications

Apart from adding to the scarce evidence base on measuring fidelity of peer support interventions, one salient aspect of the UPSIDES fidelity scale is that it was established based on both PSW and SU perspectives. Developing self-report measures for both SUs and PSWs allows future research to put a special emphasis on the multi-perspective nature of the data, considering differences and similarities in assessments of peer support contacts. The findings support the indication of a multidimensional structure of fidelity, consisting of unspecific implementation and delivery related aspects as well as peer support specific active ingredients of the intervention. Further, the correlations between the external criteria and the PSWs’ fidelity ratings are lower compared to the SUs’ ratings which we had not specifically hypothesised beforehand. These findings stimulate further research questions about the unique systematic variance within each subscale and their relationships to external criteria which is yet to be determined as inferred before elsewhere [34].

Fidelity measurement also permits comparisons across several sites, such as in the UPSIDES project [18]. The scale is available in multiple languages and was applied in different socio-economic contexts. As a next step, it can be used to investigate site-level differences in fidelity for manualised peer support. This will help to understand context-related influences on fidelity and thereby foster implementation of this complex psychosocial intervention by monitoring the quality of service delivery [8, 12].

Further, fidelity assessment helps to determine how intervention processes are associated with changes in outcome, which will in turn validate models of peer support [40] and facilitate scientific communication [24]. This highlights the essential role the fidelity scale will play in the process evaluation of the UPSIDES RCT [19].

### Practical implications

Fidelity ratings can be used to identify differences in fidelity at either the facility or individual level. Such differences support implementation improvements by learning from challenges of low fidelity sites or individuals and adopting useful strategies of high-fidelity sites or individuals [41]. The quality of peer support interventions can be enhanced by the formulation of Best Practice recommendations, which are informed by distinctions between high fidelity and low fidelity site implementation. This measure not only underscores critical elements of peer support but can also assist practitioners in consistently evaluating their work priorities and allocating resources effectively. Furthermore, it can facilitate inter-professional communication regarding the roles and responsibilities of PSWs, thus fostering organizational readiness for peer support implementation and promoting collaboration with established traditional mental health care services [42, 43].

### Recommendations for use

Based on this psychometric evaluation and subsequent considerations based on experience gained through implementing peer support in the UPSIDES project, we recommend the use of UFS-S and UFS-P for assessing fidelity to manualised UPSIDES peer support. We further recommend that high mean scores on the total scales be interpreted as indicating high manual fidelity, while lower scores indicate serious fidelity problems that should be further investigated. Part 1 (implementation) can give important insight into overall implementation of the intervention and its delivery. However, if resources are scarce, we recommend using at least the items of Part 2 (active ingredients) for fidelity assessment, as they describe specific elements of peer support. If the scores on these items are low, this is an indicator that the service may not be considerate of the most important basic principles of UPSIDES peer support. Practitioners should consider cultural nuances when applying the scale, adapting its use accordingly to maintain its validity and effectiveness in diverse settings. In addition to cultural particularities in different socio-economic or ethnic cultural groups, this also refers to the structures and organizational culture of the implementing institution. Particularly with regard to Part 1 of the scale, adjustments may be necessary, e.g. whether there is a team at all with which the PSWs could work together. However, Part 2 of the scale, which is based on the UPSIDES principles, will probably require no or very few adjustments, as the UPSIDES principles were designed for cross-cultural and cross-setting application.

## Conclusions

This study introduces a fidelity scale for manualised peer support to help facilitate process-outcome research on peer support. Both versions of the UPSIDES Fidelity Scale, UFS-S and UFS-P, have shown good reliability, as well as good construct and criterion validity. In conclusion, the scale is a pragmatic and psychometrically acceptable measure of fidelity to a manualised peer support intervention. Looking forward, further research should explore the scale’s application in non-English speaking regions and assess its longitudinal reliability to better understand its utility in sustained peer support programs globally.

## Electronic supplementary material

Below is the link to the electronic supplementary material.

Additional file 1_stages 1 and 2_ESM.pdf Provides a detailed description of procedures and sample of Stages 1 and 2 in developing the UPSIDES fidelity scale.

Additional file 2_final UPSIDES fidelity scale SU and PSW version.pdf Contains the final UPSIDES fidelity scale SU and PSW version in English, German, Luganda, Swahili, Gujarati and Hebrew.

Additional file 3_internal consistency_ESM.pdf Provides a table with the Item-Total Correlation and Cronbach’s α if item deleted for all items, as well as the alpha values for each subscale, for both UFS-S and UFS-P.

Additional file 4_factor structure.pdf Shows the results of the confirmatory factor analysis (factor loadings, correlation between factors and residuals) for both UFS-S and UFS-P.

## Data Availability

The data that support the findings of this study will be available in the repository OPARU (at https://oparu.uni-ulm.de) following an embargo until 31/12/2025, to allow for prioritised generation of research findings by members the UPSIDES consortium.

## Footnotes

1 (1) Catchment area of Ulm University’s Clinic for Psychiatry and Psychotherapy II, Ulm, Germany; (2) Department of Psychiatry at University Medical Centre Hamburg-Eppendorf and community services all over the city, Hamburg, Germany; (3) Butabika National Referral Hospital, Kampala, Uganda; (4) Department of Psychiatry and Mental Health at Muhimbili National Hospital, coordinated by Ifakara Health Institute, Dar es Salaam, Tanzania; (5) Two community rehabilitation organisations (“Kidum Proyektim Shikumiim” and “Enosh”) which provide services across the country, coordinated by the Department of Social Work at Ben Gurion University of the Negev, Be’er Sheva, Israel; (6) Hospital for Mental Health in Ahmedabad, Gujarat, coordinated by the Centre for Mental Health Law and Policy, Pune, India.

## Abbreviations

AIC: Aikaike information criterion
BIC: Bayesian information criterion
CFA: Confirmatory factor analysis
CFI: Comparative fit index
χ2: Chi-square index
DF: Degrees of freedom
M: Mean
PSW: Peer support worker
RCT: Randomised controlled trial
RMSEA: Root mean square error of approximation
SD: Standard deviation
SE: Standard error
SRMR: Standardised root mean square residual
SU: Service user
TLI: Tucker-Lewis index
UFS-S: UPSIDES Fidelity Scale (Service user version)
UFS-P: UPSIDES Fidelity Scale (Peer support worker version)
UPSIDES: Project acronym: Using Peer Support In Developing Empowering mental health Services
