# Supplementary material for: Measuring fidelity to manualised peer support for people with severe mental health conditions: development and psychometric evaluation of the UPSIDES fidelity scale
Source: BMC Psychiatry. 2024 Oct 11;24:675. doi: 10.1186/s12888-024-06081-8 (PMC11468091; doi:10.1186/s12888-024-06081-8)
Supplement: Supplementary file 1 — Additional file 1_stages 1 and 2_ESM.pdf Provides a detailed description of procedures and sample of Stages 1 and 2 in developing the UPSIDES fidelity scale. [file 12888_2024_6081_MOESM1_ESM.pdf]

Additional file 1

Description of of Stages 1 and 2: procedures and sample

Stage 1

Procedures

The initial requirement for a short, pragmatic fidelity tool that could later be used in other contexts was balanced with a rigorous, precise research effort to support ensuing data analyses, especially process evaluation [1, 2]. Since UPSIDES is a peer-delivered intervention that very much depends on building a strong, personal and trusting relationship between the peer and PSW, it is not feasible to have a supervisor sit in on peer-PSW interactions without compromising the quality of the intervention in the process. Similarly, methods of recording that might allow a supervisor to assess after-the-fact would not have been acceptable, as often very personal, private information is being shared. Based on these preconditions, in Stage 1 (Construction) the decision for a self-report measure, i.e. a pragmatic way to collect information, was made. A list of candidate items was developed by RH and BP in 2019 through a narrative literature review of: studies on fidelity measures for recovery-oriented interventions for adults with mental health conditions; studies characterising components of peer support work; and aspects specific to the UPSIDES intervention [3–6]. A 5-point Likert-scale format was chosen, ranging from 1 = “Not at all” to 5= “Very much”, in line with existing fidelity scales [1, 7]. Mean scores were used for the evaluation of fidelity, where high scores indicate high manual fidelity. Two versions of the scale were developed, to capture fidelity assessment from the perspective of the PSW and of the SU receiving peer support. The UPSIDES Fidelity Scale PSW version (UFS-P) and SU version (UFS-S) have a similar structure. Part 1 (implementation) comprises items measuring common aspects of fidelity mentioned above and are in line with the UPSIDES implementation manual [8] and the module communication of the intervention manual (Mahlke et al. 2019). Items measuring common aspects of intervention delivery in Part 1 were adapted from existing fidelity measures of mental health interventions (Lloyd-Evans et al. 2016; Chinman et al. 2016; Feely et al. 2018). Based on this, Part 1 of UFS-S was structured to assess four pre-defined domains of fidelity: receipt (how much of the PSW intervention was received), engagement (how active the SU was in the PSW relationship), enactment (how much the SU could transfer knowledge acquired from the peer support work into their daily life) and competence (the perceived interpersonal qualities and skills of the PSW). Part 1 of UFS-P was structured based on three domains: receipt, competence and

communication (cooperation with other mental health staff members).

Part 2 (active ingredients) of both UFS-S and UFS-P consists of items measuring peer support specific aspects of fidelity. These were derived from the nine principles of peer support of the UPSIDES conceptual framework, see Table A1. These nine principles capture distinct active ingredients of peer support compared to the more general items in Part 1 which revolve more around implementation and service delivery. For all principles at least one item was formulated. The principle “recovery-focused” was captured with three items, assessing the recovery components hope, control and opportunities, as described in the training manual [5]. As a result of Stage 1, UFS-S consisted of 33 items, UFS-P consisted of 31 items.

Table A1. The nine core principles of UPSIDES peer support

Principle		Description
**1**	**Mutual**<br><br>Peer support opens a space to share experiences of personal crisis and recovery.	Peer support workers have similar experiences to peer support users. The experiences of peers who give support will never be identical to those of the peers receiving support. However, peer workers in mental health settings do share some experiences with the people they work with. They understand the experience of mental health challenges, the meaning of being defined as a ‘mental patient’ in society and the confusion, loneliness, fear, and hopelessness that can ensue.
**2**	**Reciprocal**<br><br>All contribute to and gain from the relationship in a shared process of growing.	Traditional relationships between mental health professionals and the people they support are founded on the assumption of an expert (professional) and a non-expert (patient/recipient) hierarchy. Peer relationships are non-hierarchical and involve no claims to such specialist expertise but involve a sharing and exploration of different world views and the generation of solutions together. Peer support is a mutual support in a shared journey of discovery, where both people inspire and learn from each other.
**3**	**Non-directive**<br><br>Acknowledging that everybody is the expert for his/herself.	Peer support workers develop solutions together with the peers receiving support, instead of presenting solutions. In mental health services, professionals often prescribe the ‘best’ course of action for those whom they serve. Peer support is not about introducing another set of experts who offer prescriptions based on their own experience - ‘you should try this because it worked for me’. Instead, they endure difficult situations together with the peer and help people to recognise their own resources and seek their own solutions.
**4**	**Recovery- focused**<br><br>The relationship focuses on helping people to grow within and beyond what has happened	Peer support workers support the person on his/her path towards recovery, by supporting, encouraging and empowering a person to build on their personal resources and strengths, gaining hope for the future, and thinking about their personal wishes and dreams. Recovery also means focusing on more than being a patient and starting to move beyond a crisis. At the same time, peers acknowledge that recovery is a personal process and looks different for everybody.

**5**	**Empowerment**<br><br>Enable people to demand their rights and to fight stigma and discrimination.	Empowerment is all about making one's own decisions. Peer support workers support people to gain self-efficacy and to speak up for themselves. In the mental health system, users find it often difficult to enforce their own decisions for their recovery. The peer support worker can help to bridge between different perspectives.
**6**	**Strengths-based**<br><br>Recognise people's courage, strengths and skills and how they can use these to pursue their dreams.	Peer support workers emphasise the strengths and recovery progress of the peer support users. Peer support involves a relationship that is not afraid of extreme emotions and is about being with someone in their distress. But it is also about seeing within that distress the seeds of possibility and creating a fertile ground in which the person can grow. It explores what a person has gained from their experience, seeks out qualities and assets, identifies hidden achievements and celebrates what seem like the smallest steps forward.
**7**	**Inclusive & community focused**<br><br>Peers help people engage with and contribute to their communities.	Peer support workers help peer support users to have full and equal participation in the community and society. This can include navigating the mental health system as well as discovering what is outside this system. The peer support worker should always focus on the system and environments the peer support user lives in, close persons, activities the person enjoys, the living and working situation, the daily life and his/her wishes.
**8**	**Trialogue**<br><br>Incorporating different perspectives and expertises.	Peers respect diversity and difference and communicate the knowledge gained from expertise by experience to others (e.g. users, relatives, professionals, public). Bridging between the recipient, their family and mental health staff, by recognising and acknowledging the different perspectives and being a translator or advocate for their recipients to figure out the best way to work together. Peers work with mental health staff, to support recovery orientation within the service.
**9**	**Safe**<br><br>Respecting own boundaries and the boundaries of the other person.	Peer support workers and users develop a common basis of trust, based on a respectful, transparent, and shared understanding of boundaries. Supportive peer relationships involve the negotiation of what safety means to both parties. This can be achieved by discovering what makes each other feel unsafe, sharing rules of confidentiality, demonstrating compassion, authenticity and a non-judgmental attitude and acknowledging that neither of you have all the answers.

Stage 2: Refinement

Sample

Key informants were selected by RH from the UPSIDES consortium and international advisory board based on their experience with peer support and fidelity, their previous involvement in the development of the UPSIDES conceptual framework, and their study sites to represent high- and low- to middle-income settings. A group of nine key informants (female = 7, male = 2) was involved in the refinement of the scale. Members of this group were diverse in regard to career stage (four early-, two mid- and three senior- level), and location (Germany, Israel, Uganda, UK, USA, and Tanzania).

Procedures

In Phase 2 (Refinement), the initial drafts of the scale were refined through expert consultation to produce the final scales. Key informants were asked to a) rate the importance of each item to reduce the length of the scale and b) provide comments to improve the scale's construct validity, its user-friendliness and make it more feasible in the different contexts of high, middle and low-income countries. As a result, the scale was shortened by removing 5 items from the SU version and 10 items from the PSW version, resulting in a 21-item version for PSWs and a 28-item version for SUs. Further, the wording of several items was improved to be easier to understand, more precise and in line with the theoretical concept underpinning the item.

The scale underwent final language editing by a native English speaker (AC) and was then translated from English into five other languages. The translation process was based on the UPSIDES proportionate translation methodology [9]. A local translation expert panel was established at each site. Panel members were UPSIDES consortium members who are native speakers of the target language and fluent in English. These panels identified the most commonly used language at the site to avoid developing local language versions that are not used. One member of the panel conducted a forward translation of the fidelity scale from English into the local language, supported by other members of the panel as needed and feeding the result back to the panel for final approval.

References

1. Bond GR, Drake RE. Assessing the Fidelity of Evidence-Based Practices: History and Current Status of a Standardized Measurement Methodology. Adm Policy Ment Health. 2020;47:874–84. doi:10.1007/s10488-019-00991-6.
2. Moore GF, Audrey S, Barker M, Bond L, Bonell C, Hardeman W, et al. Process evaluation of complex interventions: Medical Research Council guidance. BMJ. 2015;350:h1258-h1258. doi:10.1136/bmj.h1258.
3. Chinman M, George P, Dougherty RH, Daniels AS, Ghose SS, Swift A, Delphin-Rittmon ME. Peer support services for individuals with serious mental illnesses: assessing the evidence. Psychiatr Serv. 2014;65:429–41. doi:10.1176/appi.ps.201300244.
4. Feely M, Seay KD, Lanier P, Auslander W, Kohl PL. Measuring Fidelity in Research Studies: A Field Guide to Developing a Comprehensive Fidelity Measurement System. Child Adolesc Soc Work J. 2018;35:139–52. doi:10.1007/s10560-017-0512-6.
5. Mahlke C, Nixdorf R, Repper J, Charles A, Slade M, Ryan G, et al. UPSIDES peer support training manual and workbook: Field version. UPSIDES Study; 2020.
6. Lloyd-Evans B, Bond GR, Ruud T, Ivanecka A, Gray R, Osborn D, et al. Development of a measure of model fidelity for mental health Crisis Resolution Teams. BMC Psychiatr. 2016;16:427. doi:10.1186/s12888-016-1139-4.
7. Chinman M, McCarthy S, Mitchell-Miland C, Daniels K, Youk A, Edelen M. Early stages of development of a peer specialist fidelity measure. Psychiatr Rehabil J. 2016;39:256–65. doi:10.1037/prj0000209.
8. Nakku J, Ryan G, Aslam R, Baillie D, Adler Ben-Dor I, Charles A, et al. UPSIDES Cross-Site Implementation Manual: Generic Field Version. 2023. https://www.upsides.org/multilingual-area/output/#32Manuals. Accessed 16 Feb 2024.
9. Charles A, Korde P, Newby C, Grayzman A, Hiltensperger R, Mahlke C, et al. Proportionate translation of study materials and measures in a multinational global health trial: methodology development and implementation. BMJ Open. 2022;12:e058083. doi:10.1136/bmjopen-2021-058083.
