# Supplementary material for: Measuring fidelity to manualised peer support for people with severe mental health conditions: development and psychometric evaluation of the UPSIDES fidelity scale
Source: BMC Psychiatry. 2024 Oct 11;24:675. doi: 10.1186/s12888-024-06081-8 (PMC11468091; doi:10.1186/s12888-024-06081-8)
Supplement: Supplementary file 2 — Additional file 2_final UPSIDES fidelity scale SU and PSW version.pdf Contains the final UPSIDES fidelity scale SU and PSW version in English, German, Luganda, Swahili, Gujarati and Hebrew. [file 12888_2024_6081_MOESM2_ESM.pdf]

Appendix B – Final UPSIDES fidelity scale

UFS-S (service user version) - English

	Not at all	*A little bit*	*Somewhat*	*Quite a bit*	*Very much*
1. It was easy to participate in UPSIDES peer support sessions on a regular basis (Traveling, scheduling of sessions, etc.).	○	○	○	○	○
2. The amount and duration of UPSIDES peer support was appropriate.	○	○	○	○	○
3. It was possible to contact my UPSIDES peer support worker easily when needed.	○	○	○	○	○
4. I have actively participated in UPSIDES peer support sessions.	○	○	○	○	○
5. I had no problems understanding what UPSIDES peer support is about.	○	○	○	○	○
6. My UPSIDES peer support worker was sensitive to my questions and comments.	○	○	○	○	○
7. My UPSIDES peer support worker took time to listen to me.	○	○	○	○	○
8. My UPSIDES peer support worker provided social support important to me.	○	○	○	○	○
9. My UPSIDES peer support worker showed me some skills that I have been able to transfer into my daily life.	○	○	○	○	○
10. My UPSIDES peer support worker gave me new ideas and perspectives which will remain important to me in the future.	○	○	○	○	○
11. My UPSIDES peer support worker was always well-prepared for our meetings.	○	○	○	○	○
12. My UPSIDES peer support worker was suitable / a good choice for this job.	○	○	○	○	○
13. My UPSIDES peer support worker was well-trained for this job.	○	○	○	○	○
14. My UPSIDES peer support worker's behaviour was always appropriate and professional.	○	○	○	○	○
15. My peer support worker's wellbeing was always well enough to provide support to me.	○	○	○	○	○
16. My UPSIDES peer support worker had good relationships with other clinical staff members.	○	○	○	○	○
My UPSIDES peer support worker:	*Not at all*	*A little bit*	*Somewhat*	*Quite a bit*	*Very much*
17. … served as a role model and promoted hope.	○	○	○	○	○
18. … empowered me to grow within and beyond my current mental health status.	○	○	○	○	○
19. … supported me to regain control over my life.	○	○	○	○	○
20. … helped me to reduce my isolation.	○	○	○	○	○
21. ... helped me to communicate with my family and/or MH staff about my perspective and my treatment choices	○	○	○	○	○
22. … has focussed on my strengths.	○	○	○	○	○
23. … has become a trusted companion	○	○	○	○	○
24. … did not tell me what to do but supported me to find my own solutions.	○	○	○	○	○
25. … and I have both learned and benefitted from our peer support sessions.	○	○	○	○	○
26. … has helped me to take part in my community.	○	○	○	○	○
27. … helped me to see and use new opportunities	○	○	○	○	○
28. … understood me and the situation I am in.	○	○	○	○	○

UFS-P (peer support worker version) - English

	Not at all	A little bit	Somewhat	Quite a bit	Very much
1. It was easy to provide UPSIDES peer support sessions on a regular basis. (Traveling, scheduling of sessions, etc.)	◯	◯	◯	◯	◯
2. My clients were satisfied with the amount of sessions and duration of support I could provide them in UPSIDES.	◯	◯	◯	◯	◯
3. It was possible for service users to contact me easily when needed.	◯	◯	◯	◯	◯
4. I was always well-prepared for the meetings with my clients.	◯	◯	◯	◯	◯
5. I felt that I was well-trained for this job.	◯	◯	◯	◯	◯
6. My own wellbeing was always enough to provide peer support to my clients.	◯	◯	◯	◯	◯
7. I had good relationships with other clinical staff members.	◯	◯	◯	◯	◯
8. Other mental health staff engaged with me during my work as PSW.	◯	◯	◯	◯	◯
9. Other mental health staff asked me for my point of view/ advice about mutual clients.	◯	◯	◯	◯	◯

As an UPSIDES peer support worker:	Not at all	A little bit	Somewhat	Quite a bit	Very much
10. … I have served as a role model and promoted hope.	◯	◯	◯	◯	◯
11. … I have empowered my clients to grow within and beyond their current mental health status.	◯	◯	◯	◯	◯
12. … I have enabled my clients to regain control over their lives.	◯	◯	◯	◯	◯
13. … I have helped my clients to reduce their isolation.	◯	◯	◯	◯	◯
14. … I have helped my clients to communicate with their family and/or MH staff about their perspective and treatment choices	◯	◯	◯	◯	◯
15. … I have focussed on the strengths of my clients.	◯	◯	◯	◯	◯
16. … I have become a trusted companion of my clients.	◯	◯	◯	◯	◯
17. … I did not tell my clients what to do but supported them to find their own solutions.	◯	◯	◯	◯	◯
18. … I have also learned and benefitted from the peer support sessions with my clients.	◯	◯	◯	◯	◯
19. … have helped my clients to take part in the community.	◯	◯	◯	◯	◯
20. … helped my clients to see and use new opportunities	◯	◯	◯	◯	◯
21. … I understood my clients and the situation they are in.	◯	◯	◯	◯	◯

UFS-S (service user version) - German

	Lehne stark ab	*Lehne ab*	*Weder noch*	*Stimme zu*	*Stimme voll und ganz zu*
1. Es war einfach, regelmäßig meine*n UPSIDES Peer-Begleiter*in zu treffen (Anreise, Planung, etc.).	○	○	○	○	○
2. Die Häufigkeit und Dauer der UPSIDES Peer-Begleitung war angemessen.	○	○	○	○	○
3. Falls nötig, war es einfach, meine*n UPSIDES Peer-Begleiter*in zu kontaktieren.	○	○	○	○	○
4. Ich habe aktiv an der UPSIDES Peer-Begleitung teilgenommen.	○	○	○	○	○
5. Ich hatte keine Probleme damit zu verstehen, um was es bei der UPSIDES Peer-Begleitung geht.	○	○	○	○	○
6. Mein*e UPSIDES Peer-Begleiter*in ging sensibel auf meine Fragen und Kommentare ein.	○	○	○	○	○
7. Mein*e UPSIDES Peer-Begleiter*in nahm sich Zeit, mir zuzuhören.	○	○	○	○	○
8. Mein*e UPSIDES Peer-Begleiter*in leistete soziale Unterstützung, die für mich wichtig war.	○	○	○	○	○
9. Durch meinen*meine Peer-Begleiter*in entdeckte ich Fähigkeiten, dich ich in meinen Alltag integrieren konnte.	○	○	○	○	○
10. Mein*e UPSIDES Peer-Begleiter*in zeigte mir neue Ideen und Perspektiven auf, die mir auch in Zukunft wichtig bleiben werden.	○	○	○	○	○
11. Mein*e UPSIDES Peer-Begleiter*in war auf unsere Treffen immer gut vorbereitet.	○	○	○	○	○
12. Mein*e UPSIDES Peer-Begleiter*in war eine gute Wahl für diese Arbeit.	○	○	○	○	○
13. Mein*e UPSIDES Peer-Begleiter*in war gut für diese Arbeit ausgebildet.	○	○	○	○	○
14. Das Verhalten meine*r UPSIDES Peer-Begleiter*in war immer angemessen und professionell.	○	○	○	○	○
15. Das eigene Wohlbefinden meine*r UPSIDES Peer-Begleiter*in war immer gut genug, um mir Unterstützung zu geben.	○	○	○	○	○
16. Mein*e UPSIDES Peer-Begleiter*in hatte gute Beziehungen zu anderen klinischen Mitarbeitern.	○	○	○	○	○
Mein*e UPSIDES Peer-Begleiter*in…	*Lehne stark ab*	*Lehne ab*	*Weder noch*	*Stimme zu*	*Stimme voll und ganz zu*
17. … diente mir als Vorbild und vermittelte mir Hoffnung.	○	○	○	○	○
18. …stärkte mich innerhalb und über meinen aktuellen mentalen Gesundheitsstand hinauszuwachsen.	○	○	○	○	○
19. …unterstützte mich darin, wieder Kontrolle über mein Leben zu erlangen.	○	○	○	○	○
20. … half mir dabei, meine Isolation zu verringern.	○	○	○	○	○
21. ... half mir dabei, mit meiner Familie und/oder medizinischen Fachkräften über meine Sichtweisen und meine Behandlungsentscheidungen zu sprechen.	○	○	○	○	○
22. … fokussierte sich auf meine Stärken.	○	○	○	○	○
23. … wurde zu einem*einer vertrauten Begleiter*in.	○	○	○	○	○
24. …sagte mir nicht, was zu tun war, sondern half mir, meine eigenen Lösungen zu finden.	○	○	○	○	○
25. … und ich haben beide von der Peer-Begleitung gelernt und profitiert.	○	○	○	○	○
26. … half mir, wieder am gesellschaftlichen Leben teilzunehmen.	○	○	○	○	○
27. … half mir, neue Möglichkeiten zu erkennen und zu nutzen.	○	○	○	○	○
28. …hat mich, und die Situation in der ich bin, verstanden.	○	○	○	○	○

UFS-P (peer support worker version) – German

	Lehne stark ab	Lehne ab	Weder noch	Stimme zu	Stimme voll und ganz zu
1. Es war leicht UPSIDES Peer-Begleitung regelmäßig anzubieten. (Anreise, Planung, etc.)	○	○	○	○	○
2. Mein*e Klient*in war mit der Häufigkeit und Dauer der Treffen, die ich im Rahmen von UPSIDES anbieten konnte, zufrieden.	○	○	○	○	○
3. Wenn nötig, war es für meine*n Klientin*en leicht, mich zu kontaktieren.	○	○	○	○	○
4. Für die Treffen mit meinem*r Klienten*in war ich immer gut vorbereitet.	○	○	○	○	○
5. Ich fühlte mich für die Arbeit als Peer-Begleiter*in gut ausgebildet.	○	○	○	○	○
6. Mein eigenes Wohlbefinden war immer gut genug, um meine*n Klientin*en zu unterstützen.	○	○	○	○	○
7. Ich hatte gute Beziehungen zu anderen klinischen Mitarbeitern*innen.	○	○	○	○	○
8. Während meiner Arbeit als Peer-Begleiter*in haben sich andere klinische Mitarbeiter*innen mit mir unterhalten.	○	○	○	○	○
9. Andere klinische Mitarbeiter*innen haben mich nach meiner Meinung oder einem Rat zum unserem*r gemeinsamen Klienten*in gefragt.	○	○	○	○	○

Als UPSIDES Peer-Begleiter*in…	Lehne stark ab	Lehne ab	Weder noch	Stimme zu	Stimme voll und ganz zu
10. … diente ich als Vorbild und vermittelte Hoffnung.	○	○	○	○	○
11. … stärkte ich meine*n Klientin*en, innerhalb und über ihren*seinen aktuellen mentalen Gesundheitsstand hinauszuwachsen.	○	○	○	○	○
12. … unterstützte ich meine*n Klientin*en darin, wieder Kontrolle über ihr*sein Leben zu erlangen.	○	○	○	○	○
13. … half ich meinem*r Klienten*in dabei, seine*ihre Isolation zu verringern.	○	○	○	○	○
14. … half ich meinem*r Klienten*in dabei, mit seiner*ihrer Familie und/oder Fachkräften über seine*ihre Sichtweisen und Behandlungsentscheidungen zu sprechen.	○	○	○	○	○
15. … fokussierte ich mich auf die Stärken meines*r Klienten*in.	○	○	○	○	○
16. … wurde ich zu einem*r vertrauten Begleiter*in für meine*n Klientin*en.	○	○	○	○	○
17. … sagte ich meinem*r Klienten*in nicht, was zu tun war, sondern half ihm*ihr, eigene Lösungen zu finden.	○	○	○	○	○
18. … habe auch ich von der Peer-Begleitung meines*r Klienten*in gelernt und profitiert.	○	○	○	○	○
19. … half ich meinem*r Klienten*in, am gesellschaftlichen Leben teilzunehmen.	○	○	○	○	○
20. … half ich meinem*r Klienten*in, neue Chancen und Möglichkeiten zu erkennen und zu nutzen.	○	○	○	○	○
21. … verstand ich meine*n Klientin*en und die Situation, in der sie*er sich befand.	○	○	○	○	○

UFS-S (service user version) - Luganda

	Tewali n' akatono	Kitonotono	Akatonotono	Kinene ko	Nnyo nnyini Ddala
1. Kyalikyangu bulilunaku okw'etaba mu nkungaana ez'okukubaganya ebirowoozo ez'abakozesa b'obujjanjabi era abayamba bann'abwe eza UPSIDES (okutambula, okuteekateeka enkungaana n' ebilala)	○	○	○	○	○
2. Omuwendo n'obudde okuyambagana kw'abakozesa b'obujjanjabi okwa UPSIDES bwekwatwaala bwaalibusanidde	○	○	○	○	○
3. Kyali kisoboka okuwuliziganya n'omukozi wa UPSIDES bwetukozesa obujjanjabi era annyamba mubwangu weky'ali ky'etaagisizza	○	○	○	○	○
4. Mbadde mujjumbize mukwetaba mu nkungaana eza UPSIDES ez'okukubaganya ebirowoozo ez'abakozesa b'obujjanjabi era abayamba bann'abwe	○	○	○	○	○
5. Salina buzibu okutegeera biki ebikwatagana kukukozesa obujjanjabi bw'okuyamba abalala mu UPSIDES	○	○	○	○	○
6. Omukozesa munnange ow'obujjanjabi akola omulimu gw'okunnyamba owa UPSIDES yali afaayo eri ebibuuzo ne byennamugambanga/endowooza yange	○	○	○	○	○
7. Omukozesa munnange ow'obujjanjabi akola omulimu gw'okunnyamba owa UPSIDES yatwaala obudde okumpuliriza.	○	○	○	○	○
8. Omukozesa munnange ow'obujjanjabi akola omulimu gw'okunyamba owa UPSIDES yampa obuyambi mu mbeera eyabulijjo obw'amakulu gyendi	○	○	○	○	○
9. Omukozesa munnange ow'obujjanjabi akola omulimu gw'okunyamba owa UPSIDES yandaga obumu kubukodyo bw'ensobodde okukozesa mubulamu bwange obw'abulijjo	○	○	○	○	○
10. Omukozesa munnange ow'obujjanjabi akolaomulimu gw'okunyamba owa UPSIDES yampa endowooza empya n'engeri ez'enjawulo ezijja okusigala nga zamugaso gyendi mu biseera eby'omumaaso	○	○	○	○	○
11. Omukozi munnange aw'obujjanjabi owa UPSIDES yabeeranga mwetegefu eri enkiko zaffe	○	○	○	○	○
12. Omukozesa munnange ow'obujjanjabi owa UPSIDES yali asaanidde/ eky'okusalawo ekirungi eri omulimu guno	○	○	○	○	○
13. Omukozesa munnange ow'obujjanjabi owa UPSIDES yali mutendeke bulungi eri omulimu guno	○	○	○	○	○
14. Empisa z'omukozesa munnange ow'obujjanjabi owa UPSIDES zalinga z'ezisaanidde era ngaz'ezabatendeke	○	○	○	○	○
15. Embeera y'obulamu bw'omukozesa munnange ow'obujjanjabi owa UPSIDES yalinga nnungi ekimala bulikiseera nga annyamba	○	○	○	○	○
16. Omukozesa munnange ow'obujjanjabi owa UPSIDES yalina enkolagana ennungi n'abasawo abalala	○	○	○	○	○
Omukozesa munnange ow'obujjanjabi akola omulimu gw'okunyamba owa UPSIDES …	Tewali n' akatono	Kitonotono	Akatonotono	Kinene ko	Nnyo nnyini Ddala
17. ....Yakola nga eky'okulabirako era yalinyisa essuubi	○	○	○	○	○
18. ....yampa amanyi agansobozesa okukula mundamunze n'okusukkawo kumbeera yange ey'obw'ongo gyendimu kati	○	○	○	○	○
19. ....yannyanba okuddamu okusobola okufuga obulamu bwange	○	○	○	○	○

	Tewali n' akatono	Kitonotono	Akatonotono	Kinene ko	Nnyo nnyini Ddala
20. ....yannyamba okukendezaako kukweyawula kubalala	◯	◯	◯	◯	◯
21. ....yannyamba okusobola okuwuliziganya n'abewaka awamu/ oba abasawo abajjanjaba obulwadde bw'obwongo kubikwaatagana nengeri gyendabamu ebintu n'okusalawo kugw'obujjanjabi bwange	◯	◯	◯	◯	◯
22. ....afuddeyo nnyo kubusobozi bwange/ amanyi gange	◯	◯	◯	◯	◯
23. ....afuuse munnange eyesigika	◯	◯	◯	◯	◯
24. ....teyandagira kyakukola, wabula yannyamba okuzuula eky'okuddamu eri ebizibu byange	◯	◯	◯	◯	◯
25. ....era njize awamu n'okuganyurwa mu nkungaana zaffe ez'okukubaganyizaamu ebirowoozo ez'okuyambagana	◯	◯	◯	◯	◯
26. ....annyambyeko mukweetaba/ mukwenyigira mu bikolebwa mu kitundu kyange	◯	◯	◯	◯	◯
27. ..... Annyambye ko okulaba n'okukozesa emikisa emipya	◯	◯	◯	◯	◯
28. ....yanteegera awamu n'embeera gyendimu	◯	◯	◯	◯	◯

UFS-P (peer support worker version) – Luganda

	Tewali n' akatono	*Kitonotono*	*Akatonotono*	*Kinene ko*	*Nnyo nnyini Ddala*
1. Kyalikyangu okutekawo enkungaana za UPSIDES ez'okukubaganyizaamu ebirowoozo era ez'okuyambagana bulilunaku (okutambula , okutegeka enkungaana n'ebilala)	○	○	○	○	○
2. Omukozesa ow'obujjanjabi gwe nnyamba yaali mumativu n'obunji bw'enkungaana awamu n'obudde bwenamala nga muyamba	○	○	○	○	○
3. Kyalikisoboka eri omukozesa ow'obujjanjabi gwe nnyamba okunfuna amangu lwe kyalinga ky'etaagisizza	○	○	○	○	○
4. Nalinga mwetegefu bulungi okusisinkana n'omukozesa munnange ow'obujjanjabi gwe nnyamba	○	○	○	○	○
5. Nnali nneewulira nti ntendekeddwa bulungi eri omulimu guno	○	○	○	○	○
6. Embeera y'obulamu bwange yalinga nnungi ekimala okugaba obuyambi eri mukozesa munnange ow'obujjanjabi gwe nnyamba	○	○	○	○	○
7. Nalina enkolagana ennungi n'abasawo abalala	○	○	○	○	○
8. Abasawo abalala abajjanjaba obulwadde b'obwongo be nnyigiranga wamu nange nga nkola omulimu gwange ogw'okuyamba bannange	○	○	○	○	○
9. Abasawo abalala abajjanjaba obulwadde bw'obwongo bannebuuzangako nga nkola omulimu gw'okuyamba bannange okusinziira kundowooza yange/ okw'ebuuza kubikwaatagana n'abalwadde	○	○	○	○	○

Nga omukozi wa UPSIDES akola ogw'okuyamba bakozesa banne ab'obujjanjabi…	*Tewali n' akatono*	*Kitonotono*	*Akatonotono*	*Kinene ko*	*Nnyo nnyini Ddala*
10. ... Mpeereza nga eky'okulabirako era nyimusiza essuubi	○	○	○	○	○
11. … Nyimusiza omukozesa munnange ow'obujjanjabi okukula munda n'okusukka kumbeeraye ey'obw'ongo gy'ebalimu kati	○	○	○	○	○
12. … Nsobozeseza mukozesa munnange ow'obujjanjabi okuddamu okusobola okufuga obulamu bwe	○	○	○	○	○
13. ... nnyambye mukozesa munnange ow'obujjanjabi okukendezaako ku kweyawula ku balala	○	○	○	○	○
14. ... nnyambye mukozesa munnange ow’obujjanjabi okuwuliziganya n'ab'ewaka awamu/ oba n'abasawo abajjanjaba endwadde z'obwongo kubikwaatagana n'engeri y’okukozesamu obujjanjabi	○	○	○	○	○
15. … Nfuddeyo nnyo ku busobozi bw'omukozesa wange ow'obujjanjabi	○	○	○	○	○
16. …Nfuuse munne eyeesigika.	○	○	○	○	○
17. … Omukozesa wange ow'obujjanjabi saamulagiriranga byakukola wabula namuyambako okw'ezuulira eby'okuddamu	○	○	○	○	○
18. … Era njize awamu n'okuganyurwa mu nkungaana z'etubeeramu nomukozesa wange ow'obujjanjabi.	○	○	○	○	○
19. … Nnyambyeko omukozesa w'obujjanjabi wange okusobola okw'enyigira mu bikolebwa kukyaalo.	○	○	○	○	○
20. … Nayamba omukozesa w'obujjanjabi wange okulaba n'okukozesa emikisa egiba gizze	○	○	○	○	○
21. … Nategeera omukozesa w'obujjanjabi wange awamu n'embeera gyalimu.	○	○	○	○	○

UFS-S (service user version) - Swahili

	Hapana kabisa	Kidogo sana	Kwa kiasi fulani	Kidogo	Sana
1. Ilikuwa rahisi kushiriki katika vikao vya uwezeshaji rika vya mradi wa UPSIDES mara kwa mara (Kusafiri, kupanga vipindi, nk)					
2. Muda ambao mradi wa UPSIDES wa uwezeshaji rika ulitekelezwa ulikuwa sahihi					
3. Iliwezekana kuwasiliana na mwezeshaji rika wangu wa mradi wa UPSIDES kwa urahisi wakati inahitajika.					
4. Nimeshirika kwa ukamilifu kwenye vipindi vya uwezeshaji rika kwenye mradi wa UPSIDES.					
5. Sikuwa na matatizo katika kuelewa kuhusu uwezeshaji rika kwenye mradi wa UPSIDES.					
6. Mwezeshaji rika wangu kwenye mradi wa UPSIDES alikuwa makini kwa maswali na maoni yangu.					
7. Mwezeshaji rika wangu kwenye mradi wa UPSIDES alichukua muda kunisikiliza.					
8. Mwezeshaji rika wangu kwenye mradi wa UPSIDES alitoa msaada wa kijamii ambao ulikuwa muhimu kwangu.					
9. Mwezeshaji rika wangu kwenye mradi wa UPSIDES alinionyesha ujuzi kadhaa ambao nimeweza kuhamisha katika maisha yangu ya kila siku.					
10. Mwezeshaji rika wangu kwenye mradi wa UPSIDES alinipa maoni mapya na mitazamo ambayo itabaki muhimu kwangu katika siku zijazo.					
11. Mfanyakazi wangu wa msaada wa rika kwenye mradi wa UPSIDES alikuwa amejiandaa vizuri kila wakati kwa ajili ya mikutano yetu.					
12. Mfanyakazi wangu wa Msaada wa rika kwenye mradi wa UPSIDES alikuwa mzuri/chaguo sahihi kwa kazi hii.					
13. Mfanyakazi wangu wa msaada wa rika kwenye mradi wa UPSIDES alikuwa amefundishwa vizuri kwa ajili ya kazi hii.					
14. Tabia ya mfanyakazi wangu wa msaada wa rika kwenye mradi wa UPSIDES ilikuwa sahihi na ya kitaaluma kila wakati.					
15. Ustawi wa mfanyakazi wangu wa msaada rika kwenye mradi wa UPSIDES ulikuwa unatosha mara zote kwa ajili ya kunisaidia.					
16. Mfanyakazi wangu wa msaada rika kwenye mradi wa UPSIDES alikuwa na mahusiano mazuri na wafanyakazi wengine wa kitabibu.					
Mwezeshaji rika wangu kwenye mradi wa UPSIDES…	Hapana Kabisa	Kidogo sana	Kwa kiasi fulani	Kidogo	Sana
17. … Alikuwa mfano wa kuigwa na alikuza matumaini.					
18. …. Alinipa uwezo wa kukua ndani na zaidi ya hali yangu ya afya ya akili kwa sasa.					
19. ….alinisaidia kupata tena udhibiti juu ya maisha yangu.					
20. … Alinisaidia kupunguza kutengwa kwangu.					
21. ... Amenisaidia kuwasiliana na familia yangu na wafanyakazi wa afya ya akili kuhusu mtazamo wangu na uchaguzi wa matibabu.					
22. … Amezingatia nguvu zangu.					
23. … Amekuwa rafiki wa kuaminika.					
24. … Hakuniambia nifanye nini, lakini aliniunga mkono kupata suluhisho zangu mwenyewe.					
25. … na mimi wote tumejifunza na kufaidika kutokana na vipindi vyetu vya msaada wa rika.					
26. …Amenisaidia kushiriki katika jamii yangu.					

	Hapana kabisa	Kidogo sana	Kwa kiasi fulani	Kidogo	Sana
27. … Amenisaidia kuona na kutumia fursa mpya.	○	○	○	○	○
28. … Alinielewa mimi pamoja na hali niliyokuwa nayo.	○	○	○	○	○

UFP-P (peer support worker version) - Swahili

	Hapana kabisa	*Kidogo*	*Kwa kiasi fulani*	*Kidogo*	*Sana*
1. Ilikuwa rahisi kutoa vipindi vya uwezeshaji rika kwenye mradi wa UPSIDES mara kwa mara (Kusafiri, kupanga vipindi, nk)	◯	◯	◯	◯	◯
2. Mteja wangu aliridhika na idadi ya vipindi na muda wamsaada ambao ningeweza kumpatia kwenye mradi wa UPSIDES.	◯	◯	◯	◯	◯
3. Ilikuwa inawezekana kwa mteja wangu kuwasiliana na mimi kwa urahisi wakati ilipohitajika.	◯	◯	◯	◯	◯
4. Nilikuwa nimejiandaa vya kutosha kila wakati kwa ajili ya mikutano na mteja wangu	◯	◯	◯	◯	◯
5. Nilihisi kwamba nimepata mafunzo ya kutosha kwa ajili ya hii kazi.	◯	◯	◯	◯	◯
6. Ustawi wangu ulikuwa unatosha mara zote kutoa msaada wa rika kwa mteja wangu	◯	◯	◯	◯	◯
7. Nilikuwa na mahusiano mazuri na wafanyakazi wengine wa kitabibu.	◯	◯	◯	◯	◯
8. Wafanyakazi wengine wa afya ya akili walishirikiana na mimi wakati nafanya kazi kama mwezeshaji rika.	◯	◯	◯	◯	◯
9. Wafanyakazi wengine wa afya ya akili waliomba maoni yangu au ushauri kuhusu mteja wetu wa pamoja.	◯	◯	◯	◯	◯

Kama mfanyakazi wa msaada rika kwenye mradi wa UPSIDES…	*Hapana Kabisa*	*Kidogo*	*Kwa kiasi fulani*	*Kidogo*	*Sana*
10. … Nimekuwa mfano wa kuigwa na nimekuza matumaini.	◯	◯	◯	◯	◯
11. … Nimempa uwezo mteja wangu kukua ndani na Zaidi ya hali yake ya sasa ya afya ya akili.	◯	◯	◯	◯	◯
12. … Nimemuwezesha mteja wangu kupata tena udhibiti juu ya maisha yake.	◯	◯	◯	◯	◯
13. … Nimemsaidia mteja wangu kupunguza kutengwa kwake.	◯	◯	◯	◯	◯
14. … Nimemsaidia mteja wangu kuwasiliana na familia yake au wafanyakazi wa afya ya akili kuhusu mitazamo yake na uchaguzi wa matibabu.	◯	◯	◯	◯	◯
15. … Nimejikita kwenye nguvu za mteja wangu.	◯	◯	◯	◯	◯
16. … Nimekuwa rafiki anayeaminika na mteja wangu.	◯	◯	◯	◯	◯
17. … Sikumwambia mteja wangu nini anatakiwa afanye, lakini nilimsaidia katika kutafuta suluhisho yake	◯	◯	◯	◯	◯
18. … Nimejifunza pia na kufaidikia kutokana na vipindi vya uwezeshaji rika na mteja wangu.	◯	◯	◯	◯	◯
19. … Nimemsaidia mteja wangu kushiriki katika jamii.	◯	◯	◯	◯	◯
20. … Nimemsaidia mteja wangu kuona na kutumia fursa mpya.	◯	◯	◯	◯	◯
21. … Nimemuelewa mteja wangu na hali aliyokuwa nayo.	◯	◯	◯	◯	◯

UFS-S (service user version) - Hebrew

	בכלל לא	קצת	במידה מסויימת	במידה ניכרת	מאוד
1. היה קל להשתתף במפגש תמיכת העמיתים של UPSIDES באופן קבוע (נסיעות, קביעת מפגשים וכו').	○	○	○	○	○
2. הכמות והמשך של מפגשי תמיכת העמיתים של UPSIDES היו מתאימים.	○	○	○	○	○
3. הייתה אפשרות ליצור קשר עם עובד תמיכת העמיתים שלי מטעם UPSIDES בקלות בעת הצורך.	○	○	○	○	○
4. השתתפתי באופן פעיל במפגשי תמיכת העמיתים של UPSIDES.	○	○	○	○	○
5. לא היו לי שום בעיות להבין במה תמיכת העמיתים של UPSIDES עוסקת.	○	○	○	○	○
6. עובד תמיכת העמיתים שלי מטעם UPSIDES היה רגיש לשאלות ולהערות שלי.	○	○	○	○	○
7. עובד תמיכת העמיתים שלי מטעם UPSIDES לקח את הזמן בכדי להקשיב לי.	○	○	○	○	○
8. עובד תמיכת העמיתים שלי מטעם UPSIDES סיפק תמיכה חברתית שחשובה לי.	○	○	○	○	○
9. עובד תמיכת העמיתים שלי מטעם UPSIDES הראה לי כמה כישורים שיכולתי להעביר לחיי היום יום שלי.	○	○	○	○	○
10. עובד תמיכת העמיתים שלי מטעם UPSIDES העניק לי רעיונות ופרספקטיבות חדשות שיישארו חשובות עבורי בעתיד.	○	○	○	○	○
11. עובד תמיכת העמיתים שלי מטעם UPSIDES תמיד היה מוכן היטב לפגישות שלנו.	○	○	○	○	○
12. עובד תמיכת העמיתים שלי מטעם UPSIDES היה מתאים / בחירה טובה לתפקיד הזה.	○	○	○	○	○
13. עובד תמיכת העמיתים שלי מטעם UPSIDES הוכשר היטב לתפקיד הזה.	○	○	○	○	○
14. התנהגותו של עובד תמיכת העמיתים שלי מטעם UPSIDES הייתה תמיד מותאמת ומקצועית.	○	○	○	○	○
15. הרווחה האישית של עובד תמיכת העמיתים שלי מטעם UPSIDES הייתה תמיד מספיק טובה בכדי להעניק תמיכה עבורי.	○	○	○	○	○
16. לעובד תמיכת העמיתים שלי מטעם UPSIDES היו מערכות יחסים טובות עם שאר חברי הצוות הקליני.	○	○	○	○	○
עובד תמיכת עמיתים שלי מטעם UPSIDES:	בכלל לא	קצת	במידה מסויימת	במידה ניכרת	מאוד
17. ...שימש כמודל חיקוי וקידם תקווה.	○	○	○	○	○
18. ...העצים אותי לצמוח בתוך ומעבר למצבי הנפשי העכשווי.	○	○	○	○	○
19. ...תמך בי בהחזרת השליטה על חיי.	○	○	○	○	○
20. ...עזר לי להפחית את הבידוד שלי.	○	○	○	○	○
21. ... עזר לי לתקשר עם המשפחה שלי ו/או צוות בריאות הנפש על הפרספקטיבה שלי ובחירות הטיפול שלי.	○	○	○	○	○
22. ...התמקד בחוזקות שלי.	○	○	○	○	○
23. ... הפך לבן לוויה שאני בוטח/ת בו.	○	○	○	○	○
24. ... לא אמר לי מה לעשות, אלא תמך בי במציאת פתרונות משלי.	○	○	○	○	○
25. ... ואני – למדנו שנינו והרווחנו ממפגשי תמיכת העמיתים.	○	○	○	○	○
26. ... עזר לי להשתתף בקהילה שלי.	○	○	○	○	○
27. ... עזר לי לראות ולהשתמש בהזדמנויות חדשות.	○	○	○	○	○
28. ... הבין אותי ואת המצב שבו אני נמצא.	○	○	○	○	○

UFP-P (peer support worker version) – Hebrew

		בכלל לא	קצת	במידה מסויימת	במידה ניכרת	מאוד
.1	היה קל לספק פגישות באופן סדיר (נסיעות, קביעת מפגשים, וכו').	○	○	○	○	○
.2	מקבל השירות שלי היה שבע רצון מכמות המפגשים וממשך התמיכה שיכולתי לספק לו ב- UPSIDES.	○	○	○	○	○
.3	מקבל השירות יכל ליצור עימי קשר בקלות בעת הצורך.	○	○	○	○	○
.4	תמיד הייתי מוכן היטב לפגישות עם מקבל השירות שלי.	○	○	○	○	○
.5	הרגשתי שקיבלתי הכשרה טובה לעבודה זו.	○	○	○	○	○
.6	הרווחה האישית שלי הייתה תמיד מספקת בכדי לספק תמיכת עמיתים למקבל השירות שלי.	○	○	○	○	○
.7	הייתה לי מערכת יחסים טובה עם שאר חברי הצוות הקליני.	○	○	○	○	○
.8	חברי צוות בריאות הנפש האחרים יצרו עימי אינטראקציה במהלך העבודה כעובד תמיכת עמיתים.	○	○	○	○	○
.9	חברי צוות בריאות הנפש האחרים יצרו עימי אינטראקציה במהלך העבודה כעובד תמיכת עמיתים	○	○	○	○	○
	כעובד תמיכת עמיתים מטעם ***UPSIDES*:**	בכלל לא	קצת	במידה מסויימת	במידה ניכרת	מאוד
.10	...היוותי מודל חיקוי וקידמתי תקווה.	○	○	○	○	○
.11	...העצמתי את מקבל השירות לגדול בתוך ומעבר למצב הנפשי העכשווי.	○	○	○	○	○
.12	...אפשרתי למקבל השירות שלי להשיג שליטה מחודשת על חייו.	○	○	○	○	○
.13	...עזרתי למקבל השירות שלי להפחית את הבדידות שלו.	○	○	○	○	○
.14	...סייעתי למקבל השירות שלי לתקשר עם המשפחה שלו / או צוות בריאות הנפש לגבי הפרספקטיבה שלו ובחירות הטיפול שלו.	○	○	○	○	○
.15	...התמקדתי בחוזקות של מקבל השירות שלי.	○	○	○	○	○
.16	...הפכתי לבן לוויה שבוטחים בו – עבור מקבל השירות שלי...	○	○	○	○	○
.17	...לא אמרתי למקבל השירות שלי מה לעשות, אלא תמכתי בו למצוא את הפתרונות שלו.	○	○	○	○	○
.18	...אני גם למדתי והרווחתי ממפגשי תמיכת העמיתים עם מקבל השירות שלי.	○	○	○	○	○
.19	...עזרתי למקבל השירות להשתתף בקהילה.	○	○	○	○	○
.20	...עזרתי למקבל השירות שלי להבחין ולהשתמש בהזדמנויות חדשות.	○	○	○	○	○
.21	...הבנתי את מקבל השירות שלי ואת המצב שבו הוא נמצא.	○	○	○	○	○

.

UFS-S (service user version) - Gujarati

	જરાય નહીં	થોડુંક	કંઈક અંશે	તદ્દન થોડીક	ખૂબ
1. નિયમિત ધોરણે UPSIDES પીઅર સપોર્ટ સેશન્સમાં ભાગ લેવાનું સરળ હતું (મુસાફરી, સેશન્સનું ટાઇમટેબલ, વગેરે).					
2. UPSIDES પીઅર સપોર્ટની રકમ અને અવધિ યોગ્ય હતી.					
3. જ્યારે જરૂરી હોય ત્યારે મારા UPSIDES પીઅર સપોર્ટ વોલેંટિયરનો સંપર્ક કરવો શક્ય હતું..					
4. મેં UPSIDES પીઅર સપોર્ટ સેશન્સમાં સક્રિયપણે ભાગ લીધો છે.					
5. UPSIDES પીઅર સપોર્ટ શું છે તે સમજવામાં મને કોઈ સમસ્યા નહોતી.					
6. મારા UPSIDES પીઅર સપોર્ટ વોલેંટિયર મારા પ્રશ્નો અને ટિપ્પણીઓ પ્રત્યે સંવેદનશીલ હતા.					
7. મારા UPSIDES પીઅર સપોર્ટ વોલેંટિયરે મને સાંભળવામાં સમય લીધો.					
8. મારા UPSIDES પીઅર સપોર્ટ વોલેંટિયરે મારા માટે મહત્વપૂર્ણ સામાજિક સપોર્ટ આપ્યો.					
9. મારા UPSIDES પીઅર સપોર્ટ વોલેંટિયરે મને કેટલીક કુશળતા બતાવી કે જે હું મારા દૈનિક જીવનમાં સ્થાનાંતરિત કરવામાં સક્ષમ રહું છું.					
10. મારા UPSIDES પીઅર સપોર્ટ વોલેંટિયરે મને નવા વિચારો અને દ્રષ્ટિકોણ આપ્યા જે ભવિષ્યમાં મારા માટે મહત્વપૂર્ણ રહેશે.					
11. મારા UPSIDES પીઅર સપોર્ટ કાર્યકર હંમેશાં અમારી મીટિંગ્સ માટે સારી રીતે તૈયાર રહે છે.					
12. મારા UPSIDES પીઅર સપોર્ટ કાર્યકર યોગ્ય હતા. / આ નોકરી માટે સારી પસંદગી					
13. મારા UPSIDES પીઅર સપોર્ટ કાર્યકરને આ નોકરી માટે સારી રીતે તાલીમ આપવામાં આવી હતી.					
14. મારા UPSIDES પીઅર સપોર્ટ કાર્યકરનું વર્તન હંમેશાં યોગ્ય અને વ્યવસાયિક હતું.					
15. મારું UPSIDES પીઅર સપોર્ટ વોલેંટિયરની સુખાકારી હંમેશાં મને સપોર્ટ પૂરો પાડવા માટે પૂરતી હતી.					
16. મારા UPSIDES પીઅર સપોર્ટ વોલેંટિયરના અન્ય ક્લિનિકલ સ્ટાફ સભ્યો સાથે સારા સંબંધો હતા.					
મારા યુપીએસઇડ્સ પીઅર સપોર્ટ વોલેંટિયર…(PSV)	જરાય નહીં	થોડુંક	કંઈક અંશે	તદ્દન થોડીક	ખૂબ
17. … રોલ મોડેલ તરીકે સેવા આપી અને આશાને પ્રોત્સાહન આપ્યું.					
18. … મારી વર્તમાન માનસિક સ્વાસ્થ્યની સ્થિતિની અંદર અને તેનાથી આગળ વધવા માટે મને શક્તિ આપી.					
19. … મારા જીવન ઉપર નિયંત્રણ મેળવવા માટે મને સમર્થન આપ્યું.					
20. … મારી અલગતા ઘટાડવામાં મદદ કરી.					
21. ... મારા પરિપ્રેક્ષ્ય અને મારી સારવાર પસંદગીઓ વિશે મારા કુટુંબ અને / અથવા મેન્ટલ હેલ્થ સ્ટાફ સાથે વાતચીત કરવામાં મને મદદ કરી.					
22. … મારી શક્તિ પર ધ્યાન કેન્દ્રિત કર્યું છે.					
23. … એક વિશ્વસનીય સાથી બની ગયો છે.					
24. … મને શું કરવું તે કહ્યું નહીં, પણ મારા પોતાના ઉકેલો શોધવા માટે મને ટેકો આપ્યો.					
25. … અને હું અમારા પીઅર સપોર્ટ સેશન્સથી બંને શીખી અને લાભ મેળવી શકું છું.					
26. … મારા સમુદાયમાં ભાગ લેવામાં મને મદદ કરી છે.					
27. … નવી તકો જોવા અને વાપરવામાં મને મદદ કરી					
28. … મને સમજ્યો અને હું જે પરિસ્થિતિમાં છું.					

UFP-P (peer support worker version) - Gujarati

	ક્યારેય નહીં	થોડુંક	કંઈક અંશે	તદ્દન થોડીક	ખૂબ ખૂબ
1. નિયમિત ધોરણે UPSIDES પીઅર સપોર્ટ સેશન્સ પૂરા પાડવા એ સરળ હતું. (મુસાફરી, સેશન્સ નું સમય પત્રક વગેરે)	○	○	○	○	○
2. મારા દર્દી સત્રનીરકમ અને સપોર્ટ ના સમયગાળાથી સંતુષ્ટ હતા કે જેહું તેમને UPSIDES માં પ્રદાન કરી શકું.	○	○	○	○	○
3. જ્યારે જરૂરી હોય ત્યારે દર્દી એ મારો સંપર્ક કરવો શક્ય હતો.	○	○	○	○	○
4. હું હંમેશાં મારા દર્દી સાથે ની મીટિંગ્સ માટે સારી રીતે તૈયાર હતો.	○	○	○	○	○
5. મને લાગ્યું કે હું આ કામ માટે સારી રીતે પ્રશિક્ષિત (ટ્રેઇન્ડ) છું.	○	○	○	○	○
6. મારી પોતાની સુખાકારી હંમેશાં મારા દર્દીને પીઅર સપોર્ટ પૂરા પાડવા માટે પૂરતી હતી.	○	○	○	○	○
7. મારા અન્ય ક્લિનિકલ સ્ટાફ સભ્યો સાથે સારા સંબંધો હતા	○	○	○	○	○
8. અન્ય માનસિક આરોગ્ય કર્મચારી ઓ PSV તરીકે ના મારા કાર્ય દરમિયાન મારી સાથે સંકળાયેલા છે.	○	○	○	○	○
9. અન્ય માનસિક આરોગ્ય કર્મચારીઓ એમને મ્યુચ્યુઅલ દર્દીઓ વિશેના મારામત / સલાહ માટે પૂછ્યું.	○	○	○	○	○

UPSIDES પીઅર સપોર્ટ વોલેંટિયર તરીકે…	ક્યારેય નહીં	થોડુંક	કંઈક અંશે	તદ્દન થોડીક	ખૂબ ખૂબ
10. મેં રોલ મોડેલ તરીકે કામ કર્યું છે અને આશા ને પ્રોત્સાહન આપ્યું છે.	○	○	○	○	○
11. મેં મારા દર્દી ને તેમની વર્તમાન માનસિક સ્વાસ્થ્યની સ્થિતિની અંદર અને આગળ વધવા માટે શક્તિ આપી છે.	○	○	○	○	○
12. મે મારા દર્દી ને તેમના જીવન ઉપર ફરીથી નિયંત્રણ મેળવવા માટે સક્ષમ બનાવ્યા છે.	○	○	○	○	○
13. મેં મારા દર્દી ને તેમના અલગતાને ઘટાડવા માં મદદ કરી છે.	○	○	○	○	○
14. મેં મારા દર્દી ને તેમના કુટુંબઅને / અથવા મેન્ટલ હેલ્થ સ્ટાફ સાથે તેમના પરિપ્રેક્ષ્ય અને સારવારની પસંદગી વિશે વાતચીત કરવામાં મદદ કરી છે.	○	○	○	○	○
15. મેં મારા દર્દી ની શક્તિઓ પર ધ્યાન કેન્દ્રિત કર્યું છે	○	○	○	○	○
16. હું મારા દર્દી નો વિશ્વસનીય સાથી બની ગયો છું	○	○	○	○	○
17. મેં મારા દર્દી ને શું કરવું તે કહ્યું નથી, પરંતુ તેમના પોતાના ઉકેલો શોધવા માટે તેમને ટેકો આપ્યો.	○	○	○	○	○
18. મેં મારા દર્દી સાથેના પીઅર સપોર્ટ સેશન્સથી પણ શીખ્યો અને તેનો ફાયદો મેળવ્યો	○	○	○	○	○
19. મારા દર્દી ને સમુદાયમાં ભાગલેવામાં મદદ કરી છે.	○	○	○	○	○
20. નવી તકો જોવા અને તેનો ઉપયોગ કરવામાં મારા દર્દી ને મદદ કરી.	○	○	○	○	○
21. હું મારા દર્દી અને તે જે પરિસ્થિતિ માં છે તે સમજી શક્યો છું.	○	○	○	○	○
