# Supplementary material for: Measuring fidelity to manualised peer support for people with severe mental health conditions: development and psychometric evaluation of the UPSIDES fidelity scale
Source: BMC Psychiatry. 2024 Oct 11;24:675. doi: 10.1186/s12888-024-06081-8 (PMC11468091; doi:10.1186/s12888-024-06081-8)
Supplement: Supplementary file 3 — Additional file 3_internal consistency_ESM.pdf Provides a table with the Item-Total Correlation and Cronbach’s α if item deleted for all items, as well as the alpha values for each subscale, for both UFS-S and UFS-P. [file 12888_2024_6081_MOESM3_ESM.pdf]

Additional file 3 – Internal Consistency

	Items SU fidelity scale	Item-Total Correlation	α if item deleted
	N = 244; alpha total = **.711**		
receipt	1: It was easy to participate in UPSIDES peer support sessions on a regular basis (Traveling, scheduling of sessions, etc.).	.575	.561
	2: The amount and duration of UPSIDES peer support was appropriate.	.530	.626
	3: It was possible to contact my UPSIDES peer support worker easily when needed.	.492	.669
	N = 243, alpha total = **.778**		
engagement	4: I have actively participated in UPSIDES peer support sessions.	.564	.735
	5: I had no problems understanding what UPSIDES peer support is about.	.571	.741
	6: My UPSIDES peer support worker was sensitive to my questions and comments.	.562	.738
	7: My UPSIDES peer support worker took time to listen to me.	.671	.687
	N = 243, alpha total = **.852**		
enactment	8: My UPSIDES peer support worker provided social support important to me.	.645	.863
	9: My UPSIDES peer support worker showed me some skills that I have been able to transfer into my daily life.	.765	.753
	10: My UPSIDES peer support worker gave me new ideas and perspectives which will remain important to me in the future.	.766	.753
	N = 233; alpha total = **.922**		
competence and professionalism	11: My UPSIDES peer support worker was always well-prepared for our meetings.	.796	.905
	12: My UPSIDES peer support worker was suitable / a good choice for this job.	.847	.897
	13: My UPSIDES peer support worker was well-trained for this job.	.822	.901
	14: My UPSIDES peer support worker's behaviour was always appropriate and professional.	.752	.910
	15: My UPSIDES peer support worker's wellbeing was always well enough to provide support to me.	.745	.911
	16: My UPSIDES peer support worker had good relationships with other clinical staff members.	.698	.919
	N= 242; alpha total = **.954**		
active ingredients	17: ... served as a role model and promoted hope.	.796	.950
	18: ... empowered me to grow within and beyond my current mental health status.	.828	.949
	19: ... supported me to regain control over my life.	.810	.949
	20: ... helped me to reduce my isolation.	.714	.952
	21: ... helped me to communicate with my family and/or MH staff about my perspective and my treatment choices	.672	.954
	22: ... has focussed on my strengths.	.795	.950
	23: ... has become a trusted companion.	.783	.950
	24: ... did not tell me what to do, but supported me to find my own solutions.	.770	.950
	25: ... and I have both learned and benefitted from our peer support sessions.	.832	.949
	26: ... has helped me to take part in my community.	.800	.950
	27: ... helped me to see and use new opportunities	.764	.951
	28: ... understood me and the situation I am in.	.802	.950

	Items PSW fidelity scale	Item-Total Correlation	α if item deleted
	N = 235; total alpha = **.736**		
receipt	1: It was easy to provide UPSIDES peer support sessions on a regular basis. (Traveling, scheduling of sessions, etc.)	.607	.593
	2: My client was satisfied with the amount of sessions and duration of support I could provide in UPSIDES.	.582	.632
	3: It was possible for my client to contact me easily when needed.	.501	.720
	N = 237; total alpha = **.739**		
competence	4: I was always well-prepared for the meetings with my client.	.617	.590
	5: I felt that I was well-trained for this job.	.523	.702
	6: My own wellbeing was always enough to provide peer support to my client.	.554	.666
	N = 232; total alpha = **.674**		
communication	7: I had good relationships with other clinical staff members.	.550	.522
	8: Other mental health staff engaged with me during my work as PSW.	.509	.549
	9: Other mental health staff asked me for my point of view/ advice about our mutual client.	.425	.677
	N = 231; total alpha = **.920**		
active ingredients	10: ... I have served as a role model and promoted hope.	.666	.914
	11: ... I have empowered my client to grow within and beyond his*her current mental health status.	.703	.912
	12: ... I have enabled my client to regain control over his*her life.	.760	.909
	13: ... I have helped my client to reduce his*her isolation.	.726	.911
	14: ... I have helped my client to communicate with his*her family and/or MH staff about his*her perspective and treatment choices.	.624	.916
	15: ... I have focussed on the strengths of my client.	.629	.915
	16: ... I have become a trusted companion of my client.	.709	.912
	17: ... I did not tell my client what to do, but supported him*her to find his*her own solutions.	.548	.918
	18: ... I have also learned and benefitted from the peer support sessions with my client.	.688	.913
	19: ... I have helped my client to take part in the community.	.655	.914
	20: ... I helped my client to see and use new opportunities.	.721	.911
	21: ... I understood my client and the situation he*she is in.	.629	.916
