# Supplementary material for: Measuring fidelity to manualised peer support for people with severe mental health conditions: development and psychometric evaluation of the UPSIDES fidelity scale
Source: BMC Psychiatry. 2024 Oct 11;24:675. doi: 10.1186/s12888-024-06081-8 (PMC11468091; doi:10.1186/s12888-024-06081-8)
Supplement: Supplementary file 4 — Additional file 4_factor structure.pdf Shows the results of the confirmatory factor analysis (factor loadings, correlation between factors and residuals) for both UFS-S and UFS-P. [file 12888_2024_6081_MOESM4_ESM.pdf]

## Additional file 4: Higher-order factor structure of UFS-S and UFS-P

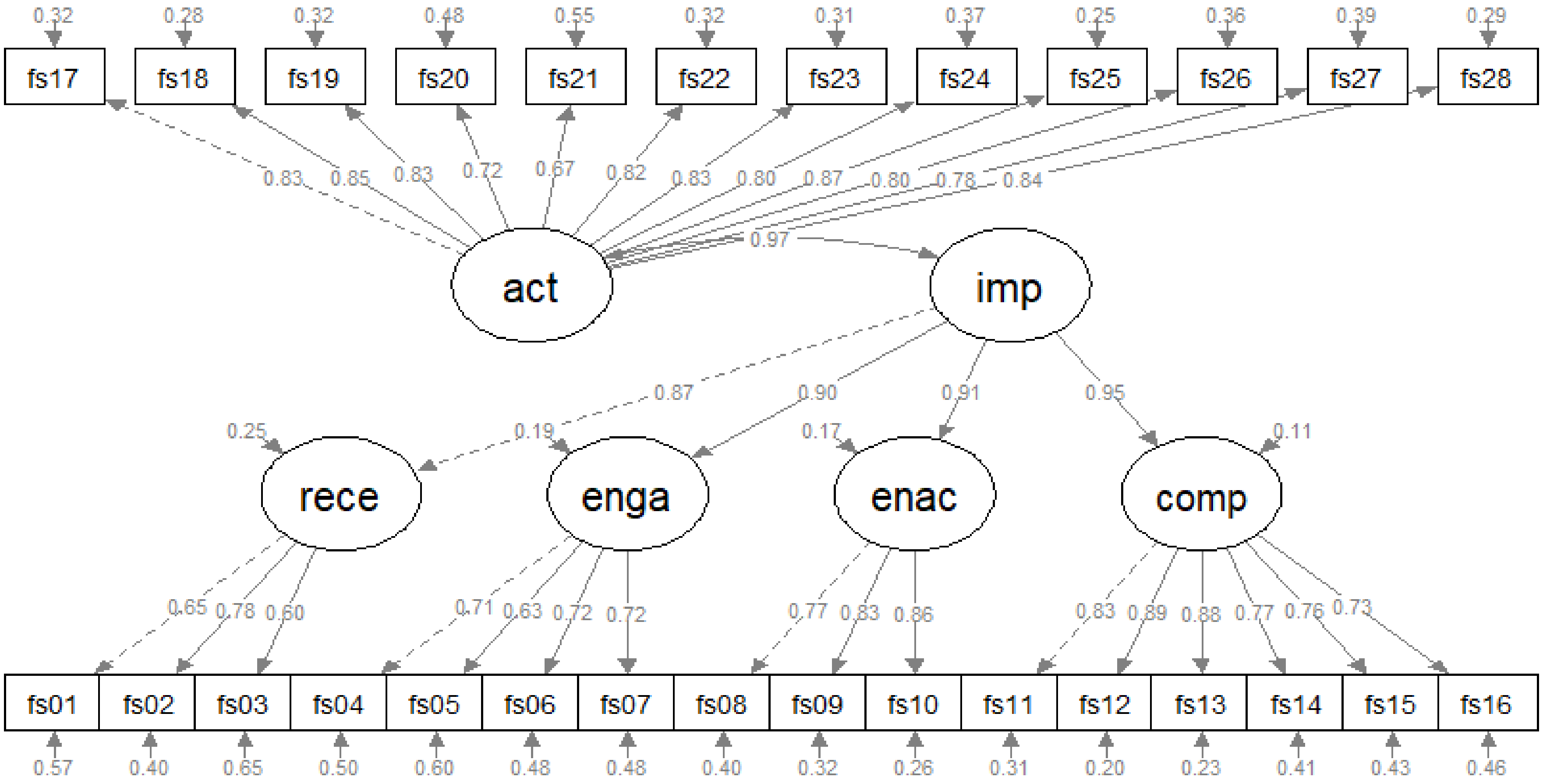


Figure 1. Higher-order factor structure for UFS-S.

Figure 1 shows factor loadings, correlation between factors and residuals for UFS-S. Abbreviations: Higher order factors: act = active ingredients; imp = implementation; factors for subscales: rece = receipt; enga = engagement; enac = enactment; comp = competence. Individual items: fs01 – fs28).

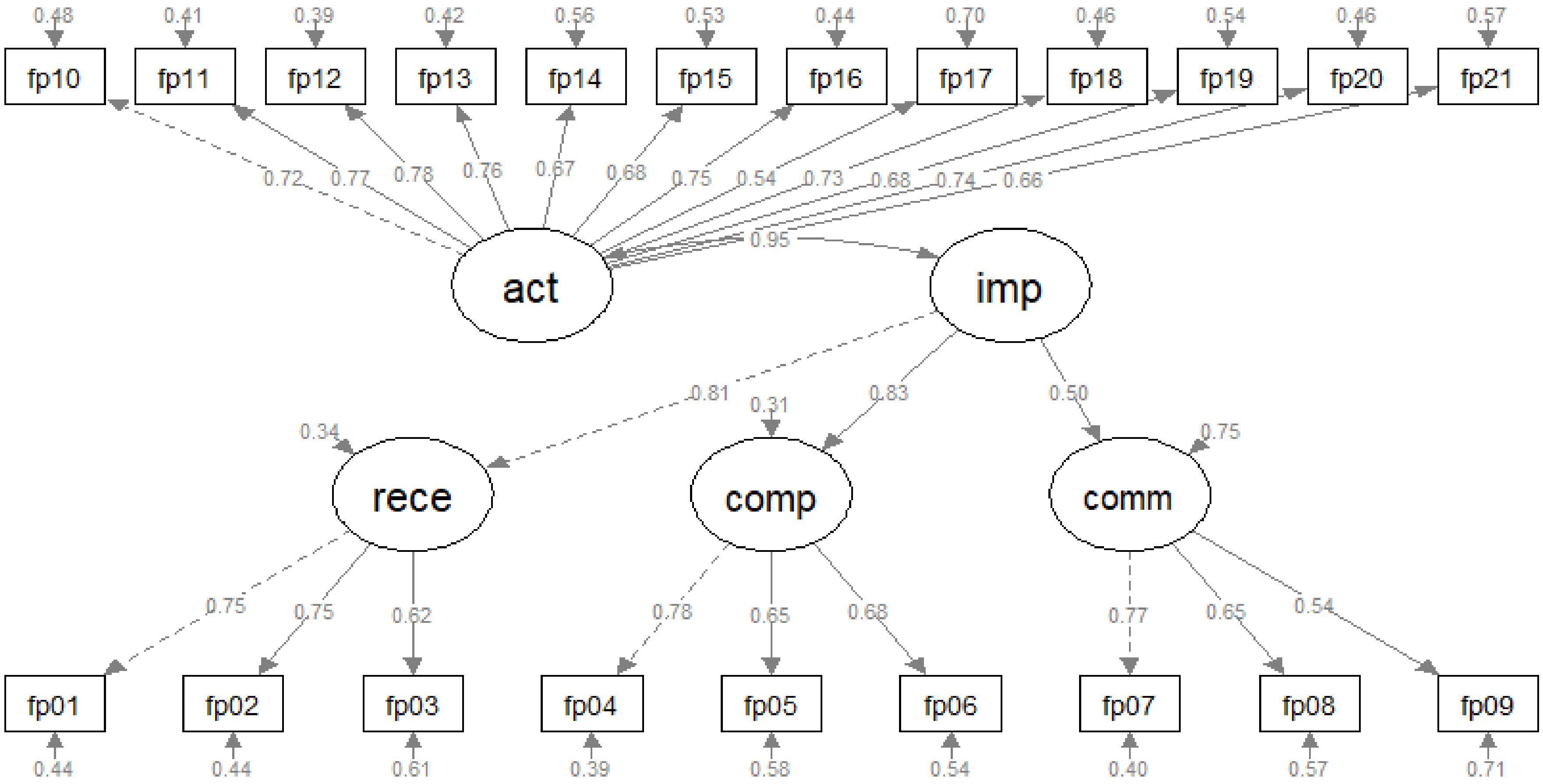


Figure 2. Higher-order factor structure for UFS-P.

Figure 1 shows factor loadings, correlation between factors and residuals for UFS-P. Abbreviations: Higher order factors: act = active ingredients; imp = implementation; factors for subscales: rece = receipt; comp = competence; comm = communication. Individual items fp01 – fp21.
